# Postmarketing reporting patterns of Candida-related events associated with bimekizumab and selective IL-17A inhibitors in psoriasis: a FAERS and JADER pharmacovigilance study

**DOI:** 10.3389/fphar.2026.1931368

**Published:** 2026-09-22

**Authors:** Hao Zhong, Yongjian Liu, Yingyu Yu, Yige Yu, Guihao Hou

**Affiliations:** 1 Department of Pharmacy, The Third Affiliated Hospital of Sun Yat-Sen University, Guangzhou, Guangdong, China; 2 Department of Pharmacy, Huizhou Central People’s Hospital, Huizhou, Guangdong, China; 3 Department of Pharmacy, Shantou Central Hospital, Shantou, Guangdong, China; 4 Department of Pharmacy, The First Affiliated Hospital of Wenzhou Medical University, Wenzhou, Zhejiang, China

**Keywords:** bimekizumab, Candida-related events, FAERS, interleukin-17, ixekizumab, JADER, pharmacovigilance, secukinumab

## Abstract

**Background:**

Candida-related events are established labelled events during IL-17 pathway inhibition. The remaining pharmacovigilance question is how *Candida* phenotypes are represented in spontaneous reports for bimekizumab and selective IL-17A inhibitors within psoriasis, while accounting for the interpretive limits of reporting-system data.

**Methods:**

We performed a phenotype-focused, dual-database disproportionality study using FAERS 2023Q4–2026Q1 as the primary source and the JADER May 2026 release as a complementary consistency source. Analyses were restricted to psoriasis-associated reports/cases. Candida-related events were the core infection phenotype; inflammatory bowel disease (IBD) and eczema/dermatitis were prespecified mechanism-related contextual domains. Reporting odds ratios (RORs) were interpreted as exploratory relative reporting contrasts. PT-level signal interpretation required at least five bimekizumab event reports (n11 ≥ 5), with n11 ≥ 10 examined as a sensitivity threshold. Age- and sex-stratified complete-case analyses and descriptive time-to-onset (TTO) sensitivity analyses were performed where data permitted.

**Results:**

The analysis included 7,850 bimekizumab and 10,971 selective IL-17A-comparator FAERS reports, and 104 bimekizumab and 890 comparator JADER cases. In FAERS, any Candida-related event was reported in 514/7,850 and 139/10,971 reports, respectively (ROR 5.46, 95% CI 4.52–6.60); JADER showed a directionally consistent reporting contrast (ROR 7.73, 95% CI 4.09–14.62). In FAERS, oral candidiasis, *Candida* infection, and oesophageal candidiasis met both n11 ≥ 5 and n11 ≥ 10 thresholds. Complete-case age and sex strata showed the same direction, although missing demographic data, particularly FAERS sex, limited interpretation. Strict TTO was evaluable for 74/514 and 45/139 Candida-positive FAERS reports.

**Conclusion:**

Candida-related phenotypes showed a reproducible disproportional reporting pattern across FAERS and JADER, whereas IBD and eczema/dermatitis findings were less consistent. The results support phenotype-focused pharmacovigilance follow-up and clinical awareness without ranking drug safety. These findings characterize spontaneous reporting patterns and should not be interpreted as estimates of incidence or comparative clinical risk.

## Introduction

Psoriasis is an IL-23/Th17/IL-17-driven immune-mediated inflammatory disease in which biologic treatment choices increasingly reflect cytokine-pathway selectivity (Lowes et al., 2007; Nestle et al., 2009; Gaffen et al., 2014; Hawkes et al., 2017). IL-17 pathway inhibitors have transformed treatment for plaque psoriasis and psoriatic arthritis, while IL-17 signalling also contributes to epithelial barrier immunity and mucosal host defence (Amatya et al., 2017; Blauvelt and Chiricozzi, 2018; Brembilla et al., 2018; Li et al., 2018). Spontaneous-reporting analyses can characterize how suspected events are reported after marketing, but they cannot supply exposure denominators or estimate clinical event probabilities.

Bimekizumab inhibits IL-17A and IL-17F, whereas secukinumab and ixekizumab selectively inhibit IL-17A. Mechanistic studies support overlapping but non-identical roles of IL-17A and IL-17F in epithelial inflammation, antimicrobial peptide induction, neutrophil-associated responses, and mucosal antifungal defence (Puel et al., 2011; Sparber and LeibundGut-Landmann, 2015; Amatya et al., 2017; Mengesha and Conti, 2017; Brembilla et al., 2018; Li et al., 2018). Here, target selectivity provides a biological framework for phenotype-focused signal description; it is not used to infer causality or a clinical safety hierarchy.

Candida-related events are neither unknown nor unexpected during IL-17 pathway inhibition. Trials, pooled safety analyses, prescribing information, and a recent broad FAERS study of bimekizumab have already documented Candida-related reporting and other adverse-event terms (Langley et al., 2014; Griffiths et al., 2015; Thaçi et al., 2015; Gordon et al., 2016; Gordon et al., 2021; Gordon et al., 2024; Blauvelt et al., 2017; Saunte et al., 2017; Reich et al., 2021a; Reich et al., 2021b; Warren et al., 2021; Schwartzman et al., 2024; DailyMed, 2025; DailyMed, 2026a; DailyMed, 2026b; Lin et al., 2026). This prior evidence motivated a narrower question about phenotype distribution, cross-database consistency, and reporting contrasts within psoriasis rather than discovery of an unrecognized adverse reaction.

Candida-related events were prespecified as the core infection phenotype because mucosal candidiasis is mechanistically linked to IL-17 blockade and clinically recognizable at the PT level. IBD was included as an established mechanism-related safety domain of IL-17 inhibition, and eczema/dermatitis was included as a clinically relevant inflammatory skin-event domain. These domains were selected *a priori* to test specificity, not to constitute a comprehensive adverse-event screen. To provide broader reporting context, we additionally summarized the ten most frequently reported PTs for each drug and database in Supplementary Table S10.

We therefore conducted a phenotype-focused, dual-database, hypothesis-generating pharmacovigilance analysis restricted to psoriasis-associated spontaneous reports. FAERS was the primary database and JADER was a complementary consistency source. The study characterized *Candida* composite and PT-level phenotypes, demographic strata where evaluable, reported TTO, serious-outcome coding, and sensitivity scenarios. Drug-to-drug RORs were treated as secondary exploratory relative reporting contrasts, not estimates of comparative clinical risk. The incremental value over prior broad bimekizumab pharmacovigilance work lies in the psoriasis restriction, FAERS-JADER triangulation, *Candida* phenotype decomposition, secondary exploratory between-drug reporting contrasts, and multiple sensitivity analyses.

## Methods

### Data sources and study design

We conducted a retrospective reporting-pattern and disproportionality analysis using FAERS and JADER (Sakaeda et al., 2013; Tsuchiya et al., 2020b; Tsuchiya et al., 2020a; Pharmaceuticals and Medical Devices Agency, 2026; U.S. Food and Drug Administration, 2026). FAERS reports from 2023Q4 through 2026Q1 captured the bimekizumab post-approval period available in the study dataset. The JADER May 2026 release served as a complementary consistency dataset. FAERS was analysed at the deduplicated report level and JADER at the reconstructed case level. The study design and analysis specifications are summarized in Supplementary Table S1.

The study was phenotype-focused rather than a comprehensive safety profiling study. Bimekizumab reports were contrasted with pooled secukinumab/ixekizumab reports to explore reporting patterns within a pharmacologically relevant comparator framework. Because spontaneous-reporting comparisons are affected by unequal market exposure, time since approval, indication and prescribing selection, geography, reporting system, stimulated reporting, notoriety and media or regulatory attention, reporter composition, and the absence of true exposure denominators, these RORs were designated exploratory relative reporting contrasts rather than clinical risk comparisons (Cutroneo et al., 2024). Drug-specific comparator analyses were retained as sensitivity analyses.

### Cohort construction and psoriasis-associated analysis set

Target-drug suspect reports/cases were identified using prespecified drug-name mappings for bimekizumab, secukinumab, and ixekizumab. Psoriasis-associated reports/cases were defined using prespecified psoriasis and psoriatic-disease indication terms, including psoriasis- and psoriatic arthritis-related terms. Reports/cases listing bimekizumab together with secukinumab and/or ixekizumab as suspect drugs were excluded to avoid ambiguous attribution between the two comparison groups. Reports/cases listing both secukinumab and ixekizumab as suspect drugs, but not bimekizumab as a suspect drug, were retained in the pooled selective IL-17A comparator but were not assigned to either single-drug comparator stratum.

In FAERS, 3,513,392 deduplicated reports from 2023Q4-2026Q1 were screened. IL-17 suspect reports numbered 52,756, of which 18,830 were psoriasis/psoriatic disease-associated. After excluding 9 mixed/multiple-target reports, the final FAERS analysis set contained 18,821 reports. In JADER, the May 2026 release was reconstructed and deduplicated at case level. IL-17 suspect cases numbered 1,190, of which 995 were psoriasis-associated. After excluding 1 mixed/multiple-target case, the final JADER analysis set contained 994 cases.

### Prespecified mechanism-related domains, PT background, and endpoints

Three mechanism-related domains were prespecified before analysis. The Candida-related domain included an any Candida-related composite and site-specific oral and oesophageal candidiasis endpoints. The IBD domain included a composite and separate ulcerative colitis and Crohn’s disease endpoints. The inflammatory skin-event domain included an eczema-like narrow endpoint and an eczema/dermatitis broad sensitivity endpoint. Preferred Term dictionaries and endpoint rules are provided in Supplementary Table S2. Separately, the ten most frequently reported PTs for bimekizumab, secukinumab, ixekizumab, and the pooled selective IL-17A comparator were tabulated within each database as descriptive context (Supplementary Table S10); this background summary was not used for comprehensive signal ranking.

The term “Candida-related events” was used for the composite because it was based on prespecified MedDRA PT matching rather than adjudicated clinical mucocutaneous candidiasis. The term “eczema-like/dermatitis-like inflammatory skin events” was used to avoid implying patient-level phenotype change, which cannot be established from spontaneous-reporting data.

### Disproportionality and exploratory reporting-contrast analyses

ROR was the prespecified primary disproportionality metric (Van Puijenbroek et al., 2002; Rothman et al., 2004). Within each endpoint and database, 2 × 2 tables contrasted endpoint-positive and endpoint-negative reports/cases for bimekizumab and pooled secukinumab/ixekizumab. RORs were reported with 95% confidence intervals (CIs), and a 0.5 continuity correction was applied when any cell was zero. For PT-level analyses, n11 denotes bimekizumab reports containing the PT. The primary interpretation threshold was n11 ≥ 5; PTs with n11 < 5 were retained descriptively but were not designated or discussed as signals. An n11 ≥ 10 sensitivity threshold assessed stability. A Candida-focused PT threshold summary is provided in Supplementary Table S11, while the complete PT-level threshold audit is provided in Supplementary Data 2. ROR magnitude was not interpreted as the magnitude of a true clinical effect, and values below 1 were not interpreted as protective effects. Variable definitions for the machine-readable PT-level output are provided in Supplementary Table S4.

PRR and IC were calculated as supportive pharmacovigilance metrics in complete report-level and PT-level analyses (Bate et al., 1998; Van Puijenbroek et al., 2002; Rothman et al., 2004; Hauben and Bate, 2009; Harpaz et al., 2013). These metrics evaluated directional coherence and did not redefine the primary ROR analysis. No disproportionality metric was interpreted as incidence, absolute clinical risk, causality, comparative safety, or clinical severity.

### Time-to-onset and serious-outcome coding

Reported time-to-onset (TTO) analyses were descriptive and restricted to Candida-positive reports with extractable, strict, non-negative therapy-start and event-onset dates. We reported the number evaluable and the proportion missing or unevaluable relative to all Candida-positive reports, medians with IQRs, and proportions with reported onset within 30 and 90 days. A common-calendar-window sensitivity analysis required both start and event dates to fall between the US bimekizumab approval date (18 October 2023) and 31 March 2026. Strict and imputation definitions are detailed in Supplementary Table S6, Supplementary Figure S5, and Supplementary Table S12.

FAERS serious-outcome fields were summarized descriptively among endpoint-positive reports using the proportion with at least one outcome code and code-level distributions. JADER serious outcomes were not harmonized because the reconstructed-case analytic extract used for this study did not retain outcome fields in a form that could be mapped consistently to FAERS OUTC categories; accordingly, JADER outcome cells are shown as NA with an explicit table note. This should not be interpreted as evidence that the source JADER system contains no outcome information. Serious-outcome coding was not interpreted as clinical severity, fatality probability, incidence, or a drug-to-drug safety comparison.

### Demographic, sensitivity, and contextual analyses

Age and sex were linked from the archived record-level demographic source. We summarized available and missing values within each database-drug stratum. FAERS age was analysed as <65 and ≥65 years; JADER age was available only in decade bands, precluding a harmonized 65-year cutoff. Sex strata were female and male. Stratified Candida-composite RORs used complete cases without imputation and were not estimated when either event-positive cell contained fewer than five reports. Sensitivity analyses also evaluated all-suspect reports, drug-specific comparators, serious-outcome reports, launch-period exclusion, the 2025Q4 reporting-volume stress test, and the PT n11 ≥ 10 threshold. The 2025Q4 exclusion was conducted after reporting-volume diagnostics (Supplementary Figure S1) and before final endpoint interpretation.

We examined whether concomitant systemic glucocorticoids, broad-spectrum antibacterials, and other immunosuppressants could be assessed. The archived report-level analytic files did not retain the complete concomitant-drug records required for a valid exposure definition; no exclusion-based concomitant-medication sensitivity analysis was therefore performed. Diabetes, immunocompromised status, and prior infection history could not be reliably distinguished from adverse-event PTs or incompletely recorded history. These limitations were acknowledged as sources of residual confounding. External context from labels, pooled trials, and case-based literature was used only to assess clinical and biological coherence, not to validate causality (Supplementary Table S7).

### Reporting standards, terminology, and software

Reporting followed transparent observational and pharmacovigilance principles, including the distinction between a signal of disproportionate reporting and clinical risk (Van Puijenbroek et al., 2002; Von Elm et al., 2007; Sakaeda et al., 2013; Cutroneo et al., 2024; U.S. Food and Drug Administration, 2026). Adverse-event terms were coded at the MedDRA Preferred Term level using version 28.1. Primary and sensitivity analyses and figures were generated with reproducible R scripts (R version 4.6.1). Microsoft Excel was used for manual review and formatting of selected source tables, and Microsoft Word was used for manuscript and table preparation.

## Results

### Cohort construction and analytic report/case characteristics

The final FAERS psoriasis-associated analysis set included 7,850 bimekizumab reports and 10,971 pooled secukinumab/ixekizumab reports; JADER included 104 and 890 reconstructed cases, respectively (Figure 1; Table 1). In FAERS, median available age was 52.0 years (IQR 41.0–61.0) and 54.0 years (IQR 41.0–64.0), with age missing in 2,936/7,850 (37.4%) and 3,610/10,971 (32.9%) reports. Sex was missing in 5,414/7,850 (69.0%) and 588/10,971 (5.4%) reports. In JADER, exact age was unavailable; decade-band age was missing in 11/104 (10.6%) and 171/890 (19.2%) cases, and sex was missing in 2/104 (1.9%) and 20/890 (2.2%) cases (Supplementary Tables S9 and S9b).

**FIGURE 1:**
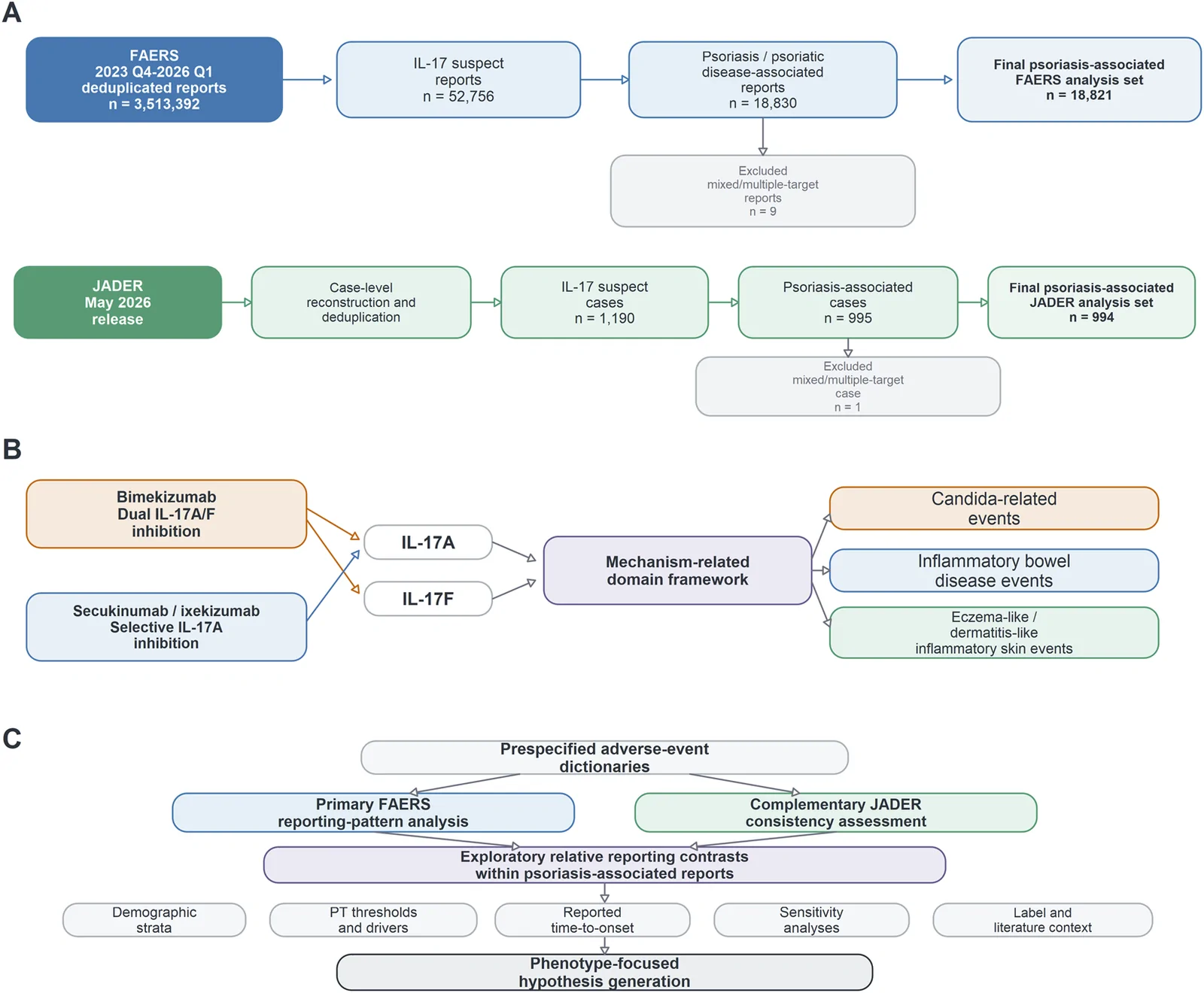
Study cohort construction and phenotype-focused analytical framework. **(A)** Cohort construction for the FAERS primary analysis and JADER complementary consistency analysis. FAERS included 2023Q4-2026Q1 deduplicated reports (n = 3,513,392), IL-17 suspect reports (n = 52,756), psoriasis/psoriatic disease-associated reports (n = 18,830), and a final analysis set (n = 18,821) after exclusion of mixed/multiple-target reports (n = 9). JADER used the May 2026 release, case-level reconstruction and deduplication, IL-17 suspect cases (n = 1,190), psoriasis-associated cases (n = 995), and a final analysis set (n = 994) after exclusion of 1 mixed/multiple-target case. **(B)** Mechanism-related framework linking target profiles to three prespecified domains: Candida-related events, IBD, and eczema/dermatitis. **(C)** Workflow from prespecified dictionaries to FAERS analysis, JADER consistency assessment, PT-level thresholding, reported TTO, sensitivity analyses, and phenotype-focused hypothesis generation. FAERS, FDA Adverse Event Reporting System; IBD, inflammatory bowel disease; JADER, Japanese Adverse Drug Event Report database; PT, Preferred Term; ROR, reporting odds ratio.

**TABLE 1:** Characteristics of the psoriasis-associated analytic FAERS reports and JADER reconstructed cases for bimekizumab and pooled selective IL-17A inhibitors.

Characteristic	FAERS		JADER	
	Bimekizumab	Secukinumab/ixekizumab	Bimekizumab	Secukinumab/ixekizumab
Analytic set size				
Final psoriasis-associated reports/cases	7,850	10,971	104	890
Report complexity				
Preferred terms per report/case, median (IQR)	2.0 (1.0–4.0)	2.0 (1.0–5.0)	1.0 (1.0–1.0)	1.0 (1.0–1.0)
Reports/cases with 1 PT	2,865 (36.5%)	4,044 (36.9%)	82 (78.8%)	692 (77.8%)
Reports/cases with 2–4 PTs	3,660 (46.6%)	4,062 (37.0%)	22 (21.2%)	190 (21.3%)
Reports/cases with 5 or more PTs	1,325 (16.9%)	2,865 (26.1%)	0 (0.0%)	8 (0.9%)
Prespecified endpoint-positive reports/cases				
Any main prespecified safety-domain endpoint-positive report/case	619 (7.9%)	382 (3.5%)	22 (21.2%)	82 (9.2%)
Serious-outcome coding context				
Any serious-outcome coding	2,916 (37.1%)	4,714 (43.0%)	NA	NA
Hospitalisation coding	825 (10.5%)	1,682 (15.3%)	NA	NA
Death coding	72 (0.9%)	280 (2.6%)	NA	NA

Abbreviations: FAERS, FDA Adverse Event Reporting System; JADER, Japanese Adverse Drug Event Report database; PT, Preferred Term; IQR, interquartile range; NA, not analysed because the reconstructed JADER extract did not retain harmonizable outcome fields. NA does not imply that the source JADER reporting system contains no outcome information.

Values are n (%) unless otherwise indicated. Percentages are column percentages using the final analytic denominator for each database-drug stratum. The pooled secukinumab/ixekizumab group represents selective IL-17A inhibition. Table 1 is descriptive; no null-hypothesis p values or clinical risk comparisons are shown.

Any prespecified domain endpoint appeared in 619/7,850 (7.9%) FAERS bimekizumab reports and 382/10,971 (3.5%) comparator reports, and in 22/104 (21.2%) and 82/890 (9.2%) JADER cases. FAERS serious-outcome coding was present in 2,916/7,850 (37.1%) and 4,714/10,971 (43.0%) reports. JADER outcomes were not reported because the reconstructed-case analytic extract did not retain harmonizable outcome fields. The most frequently reported PTs, provided as overall reporting context rather than a comprehensive safety profile, are listed by drug and database in Supplementary Table S10.

### Cross-database reporting contrasts for prespecified endpoints

Candida-related endpoints showed the clearest disproportional reporting pattern (Figure 2A; Table 2). In FAERS, any Candida-related event appeared in 514/7,850 (6.5%) bimekizumab reports and 139/10,971 (1.3%) comparator reports (ROR 5.46, 95% CI 4.52–6.60). Corresponding exploratory contrasts were observed for oral candidiasis (292/7,850 [3.7%] and 66/10,971 [0.6%]; ROR 6.38, 95% CI 4.88–8.35) and oesophageal candidiasis (69/7,850 [0.9%] and 13/10,971 [0.1%]; ROR 7.47, 95% CI 4.13–13.53). These estimates describe relative reporting odds and do not quantify relative clinical risk.

**FIGURE 2:**
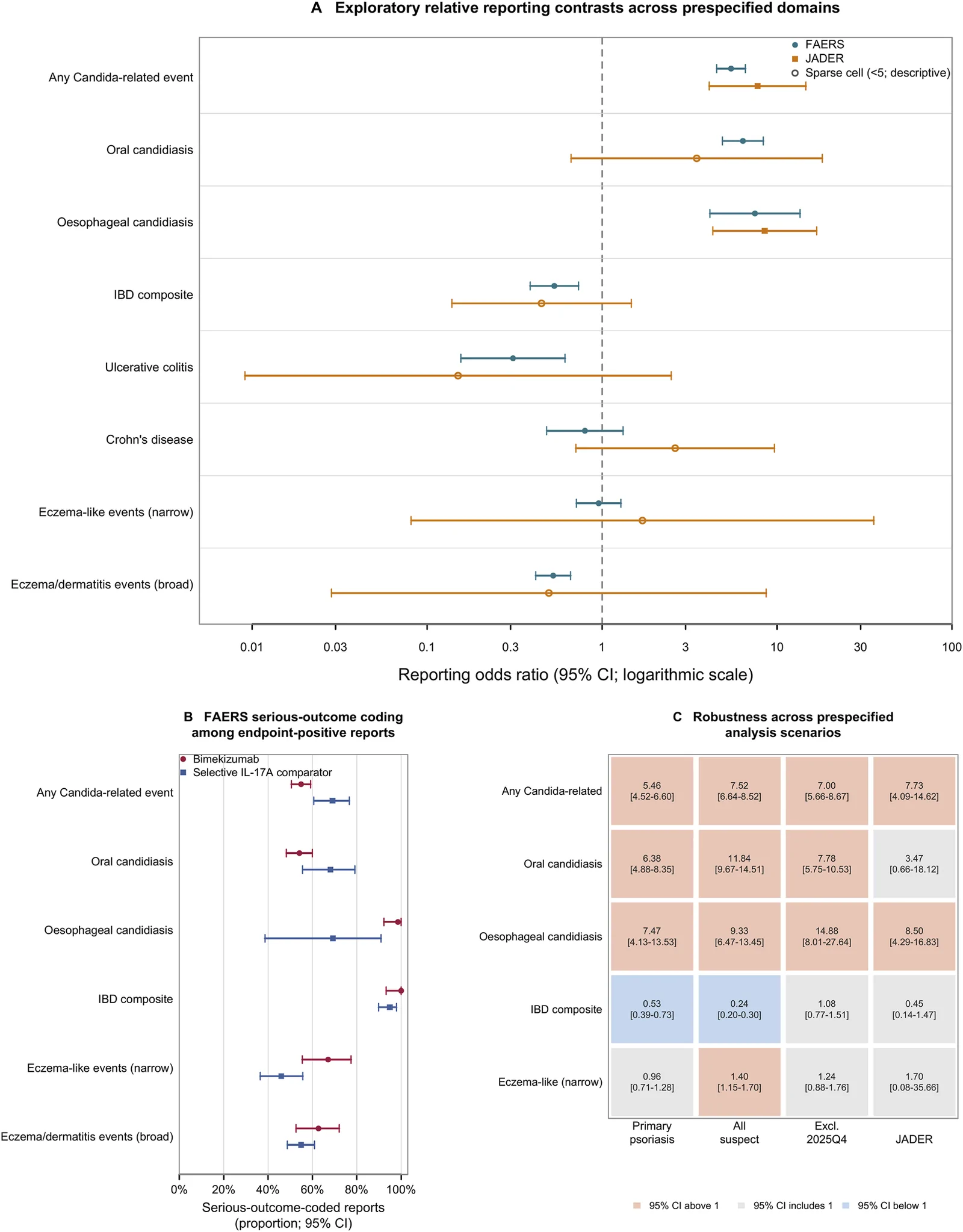
Exploratory reporting contrasts and robustness across prespecified domains. **(A)** Within-database RORs and 95% CIs for eight prespecified endpoints in FAERS and JADER. The x-axis is logarithmic and spans 0.005–100 so all CIs are fully displayed. Filled symbols denote estimates with at least five event-positive reports in both compared strata; open symbols denote a sparse event-positive cell (<5) and are descriptive. **(B)** FAERS proportions of endpoint-positive reports carrying at least one serious-outcome code, with exact binomial 95% CIs; JADER is not shown because outcome fields were not harmonized in the reconstructed-case extract. **(C)** RORs and 95% CIs across the primary psoriasis analysis, all-suspect analysis, exclusion of 2025Q4, and JADER consistency analysis. Cell colours indicate whether the 95% CI is above, includes, or is below 1. All drug-to-drug RORs are exploratory relative reporting contrasts and do not estimate comparative clinical risk. CI, confidence interval; FAERS, FDA Adverse Event Reporting System; JADER, Japanese Adverse Drug Event Report database; ROR, reporting odds ratio.

**TABLE 2:** Prespecified endpoint counts and exploratory relative reporting contrasts in FAERS and JADER.

Endpoint domain/endpoint	FAERS			JADER		
	Bimekizumab, n/N (%)	Secukinumab/ixekizumab, n/N (%)	ROR (95% CI)	Bimekizumab, n/N (%)	Secukinumab/ixekizumab, n/N (%)	ROR (95% CI)
Candida-related events						
Any Candida-related event	514/7,850 (6.5%)	139/10,971 (1.3%)	5.46 (4.52–6.60)	19/104 (18.3%)	25/890 (2.8%)	7.73 (4.09–14.62)
Oral candidiasis	292/7,850 (3.7%)	66/10,971 (0.6%)	6.38 (4.88–8.35)	2/104 (1.9%)	5/890 (0.6%)	3.47 (0.66–18.12)
Oesophageal candidiasis	69/7,850 (0.9%)	13/10,971 (0.1%)	7.47 (4.13–13.53)	17/104 (16.3%)	20/890 (2.2%)	8.50 (4.29–16.83)
Inflammatory bowel disease events						
IBD composite	53/7,850 (0.7%)	138/10,971 (1.3%)	0.53 (0.39–0.73)	3/104 (2.9%)	55/890 (6.2%)	0.45 (0.14–1.47)
Ulcerative colitis	10/7,850 (0.1%)	45/10,971 (0.4%)	0.31 (0.16–0.61)	0/104 (0.0%)	27/890 (3.0%)	0.15 (0.01–2.48)
Crohn’s disease	24/7,850 (0.3%)	42/10,971 (0.4%)	0.80 (0.48–1.32)	3/104 (2.9%)	10/890 (1.1%)	2.61 (0.71–9.65)
Eczema-like/dermatitis-like inflammatory skin events						
Eczema-like events, narrow	76/7,850 (1.0%)	111/10,971 (1.0%)	0.96 (0.71–1.28)	0/104 (0.0%)	2/890 (0.2%)	1.70 (0.08–35.66)
Eczema/dermatitis events, broad sensitivity	102/7,850 (1.3%)	268/10,971 (2.4%)	0.53 (0.42–0.66)	0/104 (0.0%)	8/890 (0.9%)	0.50 (0.03–8.67)

Abbreviations: ROR, reporting odds ratio; CI, confidence interval; FAERS, FDA Adverse Event Reporting System; JADER, Japanese Adverse Drug Event Report database; IBD, inflammatory bowel disease; PRR, proportional reporting ratio; IC, information component.

Endpoint-positive reports/cases are shown as n/N (%) with within-database RORs (95% CIs). FAERS is the primary reporting-pattern analysis; JADER is a complementary consistency analysis. Denominators are the final psoriasis-associated analytic report/case counts in Table 1.

RORs and 95% CIs were calculated from within-database 2 × 2 tables. ROR is a reporting-disproportionality metric and should not be interpreted as incidence, causality, safety ranking, or comparative clinical risk. A 0.5 continuity correction was applied when any cell was zero. PT-level analyses required n11 ≥ 5 for primary signal interpretation; n11 < 5 was descriptive only, and n11 ≥ 10 was examined as a sensitivity threshold. PRR and IC are supportive metrics reported in the Supplementary Materials.

JADER showed a directionally consistent *Candida* reporting contrast despite the small bimekizumab stratum. Any Candida-related event appeared in 19/104 (18.3%) and 25/890 (2.8%) cases (ROR 7.73, 95% CI 4.09–14.62), and oesophageal candidiasis in 17/104 (16.3%) and 20/890 (2.2%) cases (ROR 8.50, 95% CI 4.29–16.83). Oral candidiasis was sparse (2/104 [1.9%] and 5/890 [0.6%]; ROR 3.47, 95% CI 0.66–18.12) and was retained only as a descriptive, imprecise contrast.

In FAERS, the IBD composite was reported in 53/7,850 (0.7%) and 138/10,971 (1.3%) reports (ROR 0.53, 95% CI 0.39–0.73). Ulcerative colitis had lower relative reporting odds in the bimekizumab stratum (ROR 0.31, 95% CI 0.16–0.61), whereas the Crohn’s disease CI included 1 (ROR 0.80, 95% CI 0.48–1.32). JADER IBD estimates were imprecise. These values were not interpreted as protection, lower incidence, or a favourable safety ranking.

FAERS eczema-like narrow reporting proportions were similar (76/7,850 [1.0%] and 111/10,971 [1.0%]; ROR 0.96, 95% CI 0.71–1.28), while the broad eczema/dermatitis endpoint showed lower relative reporting odds (102/7,850 [1.3%] and 268/10,971 [2.4%]; ROR 0.53, 95% CI 0.42–0.66). JADER skin-event cells were sparse and descriptive. The pattern was sensitivity-dependent and was not used to infer comparative clinical safety.

### FAERS serious-outcome coding and robustness analyses

Figure 2B provides descriptive FAERS serious-outcome coding across the *Candida* composite, oral and oesophageal candidiasis, the IBD composite, and the narrow and broad eczema/dermatitis endpoints. For the *Candida* composite, at least one serious-outcome code was present in 282/514 (54.9%; 95% CI 50.4–59.2) bimekizumab and 96/139 (69.1%; 95% CI 60.7–76.6) comparator reports. These report-field summaries do not establish comparative clinical severity. The corresponding endpoint-level proportions and code-level distributions are provided in Supplementary Figure S6.

The *Candida* reporting contrast persisted across prespecified sensitivity scenarios (Figure 2C; Supplementary Figures S2 and S3; Supplementary Table S5). After excluding the 2025Q4 bimekizumab reporting-volume peak, RORs were 7.00 for any Candida-related event, 7.78 for oral candidiasis, and 14.88 for oesophageal candidiasis. Non-Candida endpoints shifted more across scenarios. These analyses support stability of the reporting pattern, not stability of a clinical treatment effect.

### Candida-specific reporting patterns and supportive analyses

Across all 10 quarters from 2023Q4 to 2026Q1, the reporting proportion of any Candida-related event among FAERS psoriasis-associated reports was higher for bimekizumab than for the pooled secukinumab/ixekizumab comparator (Figure 3A). These reporting proportions do not estimate incidence.

**FIGURE 3:**
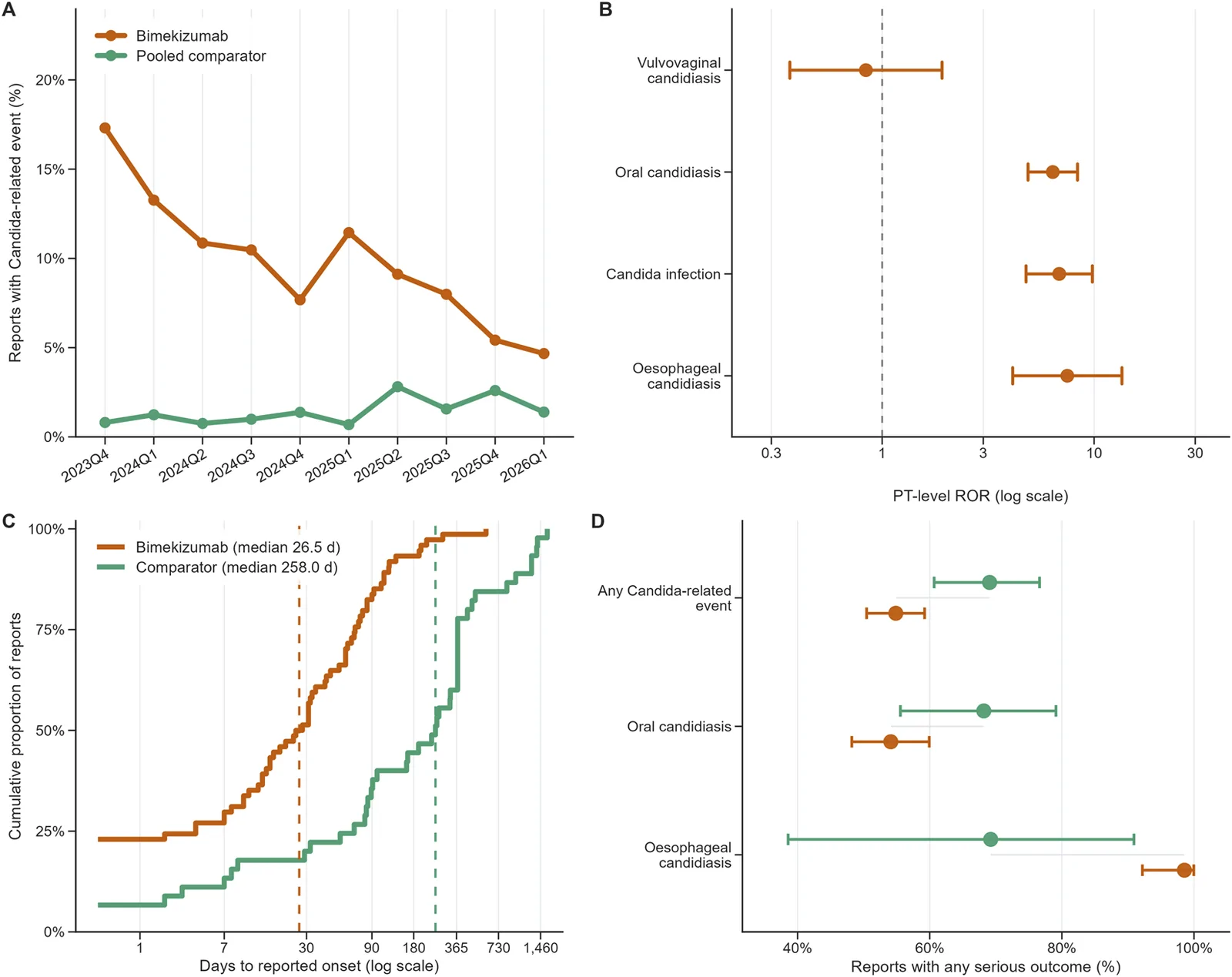
Candida-specific characterization in FAERS psoriasis-associated reports. **(A)** Quarterly reporting proportions of any Candida-related event among FAERS psoriasis-associated reports from 2023Q4 to 2026Q1, comparing bimekizumab with the pooled secukinumab/ixekizumab comparator. For each quarter and group, the numerator is the number of reports containing any Candida-related event and the denominator is all psoriasis-associated reports in that quarter and group. Across the 10 quarters, the respective totals are 514/7,850 and 139/10,971; these proportions are not incidence rates. **(B)** PT-level RORs and 95% CIs for four Candida-related PTs. The primary PT interpretation threshold was n11 ≥ 5; PTs with n11 < 5 were descriptive only. An n11 ≥ 10 sensitivity threshold retained oral candidiasis, Candida infection, and oesophageal candidiasis; vulvovaginal candidiasis (n11 = 9) did not meet the sensitivity threshold. **(C)** Empirical cumulative distribution of reported TTO for reports with strict, non-negative therapy-start and event dates (bimekizumab n = 74; comparator n = 45). The x-axis uses a log1p scale; medians are descriptive and do not establish clinical onset-speed differences. **(D)** Proportions of Candida-positive FAERS reports coded with any serious outcome, with exact binomial 95% CIs; outcome coding is not comparative clinical severity. CI, confidence interval; PT, Preferred Term; ROR, reporting odds ratio; TTO, time-to-onset.

Figure 3B resolves the *Candida* composite into four displayed FAERS PTs. Oral candidiasis (n11 = 292; ROR 6.38, 95% CI 4.88–8.35), *Candida* infection (n11 = 173; ROR 6.84, 95% CI 4.77–9.81), and oesophageal candidiasis (n11 = 69; ROR 7.47, 95% CI 4.13–13.53) showed disproportionate reporting and met both the primary n11 ≥ 5 and sensitivity n11 ≥ 10 thresholds. Vulvovaginal candidiasis (n11 = 9; ROR 0.84, 95% CI 0.37–1.92) met the primary count threshold but not the n11 ≥ 10 sensitivity threshold, and its confidence interval included 1. PTs with n11 < 5 were descriptive only. The Candida-focused threshold summary is provided in Supplementary Table S11, with the complete PT-level audit in Supplementary Data 2. In JADER, oesophageal candidiasis met both thresholds (n11 = 17), whereas oral candidiasis (n11 = 2) was descriptive only. The expanded PT-level reporting landscape is shown in Supplementary Figure S4.

Strict, non-negative TTO was evaluable in 74/514 (14.4%) bimekizumab and 45/139 (32.4%) comparator Candida-positive FAERS reports; 440/514 (85.6%) and 94/139 (67.6%), respectively, were missing or unevaluable. Median reported TTO was 26.5 days (IQR 4–68) and 258 days (IQR 67–372). Among evaluable reports, 38/74 (51.4%) and 9/45 (20.0%) had reported onset within 30 days, and 62/74 (83.8%) and 16/45 (35.6%) within 90 days. In the common calendar window, 55 bimekizumab reports had a median TTO of 24 days (IQR 0–64), whereas only five comparator reports were evaluable (median 89 days, IQR 7–98), precluding a stable between-stratum timing interpretation (Figure 3C; Supplementary Table S12).

Figure 3D narrows the serious-outcome summary to Candida-positive FAERS reports. At least one serious-outcome code was present in 282/514 (54.9%) versus 96/139 (69.1%) reports for any Candida-related event, 158/292 (54.1%) versus 45/66 (68.2%) for oral candidiasis, and 68/69 (98.6%) versus 9/13 (69.2%) for oesophageal candidiasis in the bimekizumab and comparator strata, respectively. These proportions and their exact binomial 95% CIs are descriptive report-field summaries, not measures of comparative clinical severity.

PRR and IC were directionally coherent with the report-level and eligible PT-level ROR findings (Supplementary Table S3; Supplementary Data 1). The n11 ≥ 10 sensitivity threshold retained the three principal positive FAERS *Candida* PT drivers and the JADER oesophageal-candidiasis driver. ROR, PRR, and IC remained reporting-disproportionality measures and were not combined into a safety ranking.

Complete-case sex-stratified *Candida* RORs had the same direction in FAERS women (7.43, 95% CI 5.66–9.77) and men (7.80, 95% CI 5.24–11.61), and in JADER women (4.66, 95% CI 1.53–14.17) and men (10.29, 95% CI 4.48–23.64). FAERS age-stratified RORs were 4.82 (95% CI 3.67–6.32) for <65 years and 6.23 (95% CI 3.79–10.26) for ≥65 years (Supplementary Table S9c). These exploratory strata were not adjusted, and the high FAERS sex-missingness, especially for bimekizumab, limits comparison across strata.

### Label-anchored contextualization of reporting patterns

Label mapping confirmed that the three prespecified domains are clinically relevant across IL-17 inhibitors (Figure 4; Supplementary Table S8). Candida-event frequency is described more prominently in bimekizumab prescribing information, while oral candidiasis is generally labelled as uncommon in the reviewed prescribing information for the selective IL-17A inhibitors. Concordance between labelling and a spontaneous-reporting pattern provides context but does not validate causality or quantify event probability.

**FIGURE 4:**
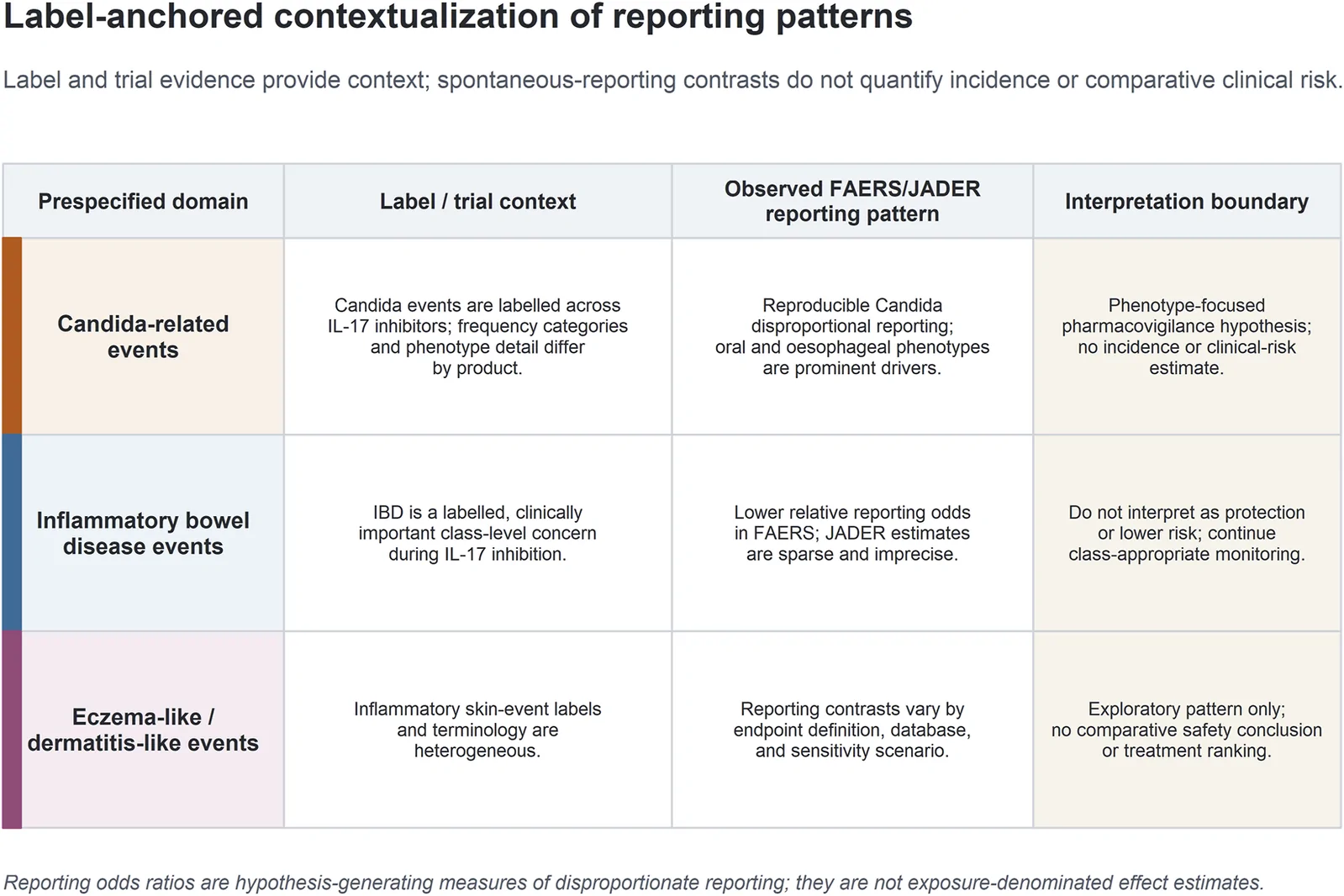
Label-anchored contextualization of prespecified reporting domains. FDA label and pooled-trial context are aligned with FAERS/JADER reporting patterns for the three prespecified domains. The matrix separates labelled clinical context from spontaneous-reporting observations and hypothesis-generating interpretation. It does not quantify event rates, establish causality, or rank comparative safety. FDA label details are provided in Supplementary Table S8.

IBD is a clinically important class-level labelled concern. The observed RORs describe reporting proportions within the selected databases and do not show whether bimekizumab has lower, equal, or higher clinical IBD risk than other IL-17 inhibitors. Continued class-appropriate monitoring remains warranted irrespective of the exploratory reporting contrast.

Inflammatory skin-event labels were heterogeneous, and the eczema/dermatitis reporting contrasts varied by definition and database. The most conservative interpretation is that these exploratory data do not support a stable database-level differential reporting pattern; they do not support a clinical safety comparison.

## Discussion

This phenotype-focused, dual-database study identified a reproducible Candida-related disproportional reporting pattern within psoriasis-associated reports. FAERS provided the primary evidence, JADER supplied a directionally consistent but smaller external dataset, and sensitivity scenarios supported the persistence of the main *Candida* contrast. PT-level decomposition localized the pattern principally to oral candidiasis, *Candida* infection, and oesophageal candidiasis. IBD and eczema/dermatitis contrasts were less consistent and were not used to rank clinical safety.

A recent FAERS study performed a broad all-event bimekizumab signal scan and documented candidiasis among frequently reported events (Lin et al., 2026). The present work addresses a different, incremental question: it restricts the cohort to psoriasis, triangulates FAERS with JADER, characterizes *Candida* phenotypes at the PT level, evaluates secondary exploratory between-drug reporting contrasts, applies count-threshold and multiple sensitivity analyses, examines reported TTO, and explicitly compares cross-database agreement and divergence. The contribution is therefore phenotype-focused and hypothesis-generating rather than a claim of novelty for the *Candida* association or a comprehensive safety profile.

The *Candida* pattern is biologically coherent with the role of IL-17 signalling in mucosal antifungal defence (Puel et al., 2011; Sparber and LeibundGut-Landmann, 2015; Amatya et al., 2017; Mengesha and Conti, 2017; Saunte et al., 2017; Li et al., 2018; McInnes et al., 2023). IL-17A and IL-17F contribute to barrier-site immunity, antimicrobial peptide induction, and neutrophil-linked host defence. Dual IL-17A/F blockade may plausibly influence the observed reporting phenotype, but spontaneous reports cannot establish molecular causality or translate ROR magnitude into clinical effect size.

The *Candida* phenotype distribution differed between databases: oral candidiasis accounted for a larger proportion of bimekizumab reports in FAERS, whereas oesophageal candidiasis predominated in the much smaller JADER stratum. Plausible explanations include database size and case counts, different reporting sources and reporting rules, variation in diagnostic work-up (including endoscopy), MedDRA coding practices, regional prescribing and patient composition, time since launch, and stimulated reporting. With only 104 JADER bimekizumab cases, site-specific proportions are especially unstable. The discordance therefore cannot be interpreted as a true regional difference in the incidence or anatomical distribution of candidiasis.

The marked TTO contrast also requires caution. Bimekizumab entered the market later and consequently had a shorter observable window, creating right truncation and greater sensitivity to stimulated early post-launch reporting. The strata may differ in exposure duration, date completeness, and the composition of susceptible patients; early events among susceptible individuals and survivor or depletion-of-susceptibles effects among long-term comparator users may also contribute. The common-window sensitivity analysis left only five evaluable comparator reports, and 85.6% of bimekizumab Candida-positive reports lacked evaluable strict dates. Accordingly, the observed medians of 26.5 and 258 days should not be interpreted as a true difference in clinical onset speed.

The clinical implication is focused awareness of labelled *Candida* manifestations rather than treatment ranking. Clinicians may continue counselling and monitoring for oral or genital *Candida* symptoms, dysphagia, and odynophagia in accordance with prescribing information and individual clinical context. IBD remains an important class-level concern, and the absence of a stable positive reporting contrast cannot be interpreted as absence of clinical risk. The selected domains help define the boundary of the *Candida* reporting pattern, but this study is not a comprehensive safety profiling analysis.

Limitations inherent to spontaneous reporting remain substantial. FAERS and JADER lack reliable exposure denominators and are affected by under-reporting, duplicate or stimulated reporting, notoriety and media or regulatory attention, different time on market, indication and channeling, geography, reporter composition, and missing data (Cutroneo et al., 2024). The archived analysis files did not retain complete concomitant-drug records, so systemic glucocorticoids, broad-spectrum antibacterials, and other immunosuppressants could not be consistently characterized or excluded. Diabetes, immunocompromised status, prior infection, and other patient-level factors were incompletely identifiable, leaving residual confounding. The demographic analyses were complete-case and unadjusted; FAERS sex was particularly incomplete. JADER bimekizumab cells were sparse, and harmonized JADER serious outcomes were unavailable in the reconstructed extract. *Candida* PTs were not clinically adjudicated, and TTO depended on incomplete dates. No analysis fully adjusted confounding.

Exposure-denominated electronic health records, claims data, registries, and prospective cohorts are needed to estimate event frequencies, adjudicate phenotypes, adjust patient-level covariates, and test whether the reporting hypotheses correspond to clinical differences. Until then, between-drug RORs should be interpreted only as secondary exploratory reporting contrasts, and no drug safety ranking is warranted. These findings characterize spontaneous reporting patterns and should not be interpreted as estimates of incidence or comparative clinical risk.

## Conclusion

In psoriasis-associated spontaneous reports, Candida-related events showed a reproducible pharmacovigilance signal and phenotype pattern across FAERS and JADER, with oral candidiasis, *Candida* infection, and oesophageal candidiasis as the main eligible PT-level drivers. IBD and eczema/dermatitis contrasts were less consistent and do not establish favourable or unfavourable comparative safety. The analysis is hypothesis-generating and supports phenotype-focused follow-up without ranking the drugs. These findings characterize spontaneous reporting patterns and should not be interpreted as estimates of incidence or comparative clinical risk.

## Data Availability

The raw data supporting the conclusions of this article will be made available by the authors, without undue reservation.
