# Supplementary material for: Postmarketing reporting patterns of Candida-related events associated with bimekizumab and selective IL-17A inhibitors in psoriasis: a FAERS and JADER pharmacovigilance study
Source: Front Pharmacol. 2026 Sep 22;17:1931368. doi: 10.3389/fphar.2026.1931368 (PMC13638565; doi:10.3389/fphar.2026.1931368)
Supplement: Supplementary file 1 [file DataSheet1.pdf]

Figure S1. FAERS reporting-volume and indication-composition diagnostics. (A) Quarterly target-drug suspect reports in FAERS. The shaded band marks the 2025Q4 bimekizumab reporting-volume peak. (B) Psoriasis-associated reports among target-drug suspect reports. Fractions represent database reporting proportions rather than incidence rates. The 2025Q4 bimekizumab reporting-volume peak was evaluated in a quarter-exclusion sensitivity analysis.

Figure S2. All-suspect report-level ROR sensitivity analysis. Estimates were calculated irrespective of reported indication and interpreted as sensitivity results rather than primary comparative estimates because indication composition differed between drug groups. Points represent RORs and horizontal bars represent 95% CIs; right-side text shows bimekizumab versus pooled comparator endpoint-positive reports/cases. Sparse JADER estimates were interpreted as directional checks rather than precise comparative estimates. A 0.5 continuity correction was applied when any 2 x 2 table cell was zero. BIM, bimekizumab; comp., pooled secukinumab/ixekizumab comparator; IBD, inflammatory bowel disease; ROR, reporting odds ratio.

Figure S3. FAERS ROR sensitivity matrix for prespecified endpoints. Cell text shows raw ROR values and colour maps log2(ROR), with blue indicating lower relative reporting, near-white indicating ROR values close to 1, and red indicating higher relative reporting. The 2025Q4 exclusion analysis was used as a reporting-volume stress test because of a bimekizumab reporting-volume peak in that quarter. Complete confidence intervals and cell counts are provided in Supplementary Table S5. Isolated shifts in sparse endpoints were interpreted cautiously.

Figure S4. Expanded PT-level ROR landscape of prespecified safety domains in FAERS psoriasis-associated reports. Selected top-reported Preferred Terms within each domain are shown. Points represent PT-level RORs and horizontal bars represent 95% CIs; point size represents PT-positive reports. PT-level analyses were used to identify signal drivers within prespecified domains and were interpreted as exploratory supportive analyses. Sparse PT-level estimates with wide confidence intervals were interpreted cautiously. Complete PT-level results are provided in Supplementary Data 1; variable definitions are provided in Supplementary Table S4. PT, Preferred Term; ROR, reporting odds ratio.

Figure S5. Reported time-to-onset sensitivity analysis comparing strict and imputed onset

definitions. Points show median reported days to onset, horizontal bars show IQRs, and point size represents reports with evaluable TTO. TTO analyses were restricted to reports with extractable and non-negative therapy-start and event-onset dates. These summaries describe reported onset timing among evaluable reports and should not be interpreted as comparative clinical timing estimates. Sparse JADER summaries were interpreted descriptively. IQR, interquartile range; TTO, time-to-onset.

Figure S6. Serious-outcome coding context in FAERS psoriasis-associated endpoint-positive reports. (A) Proportions of endpoint-positive reports with at least one serious-outcome code, with binomial 95% CIs; point size represents endpoint-positive reports. (B) Code-level distribution of serious outcomes. Multiple serious-outcome codes can occur in one report; therefore, panel B summarizes code-level shares rather than mutually exclusive report-level outcomes. Serious-outcome coding reflects spontaneous-report outcome fields and should not be interpreted as comparative clinical severity, event fatality, or incidence. CI, confidence interval.

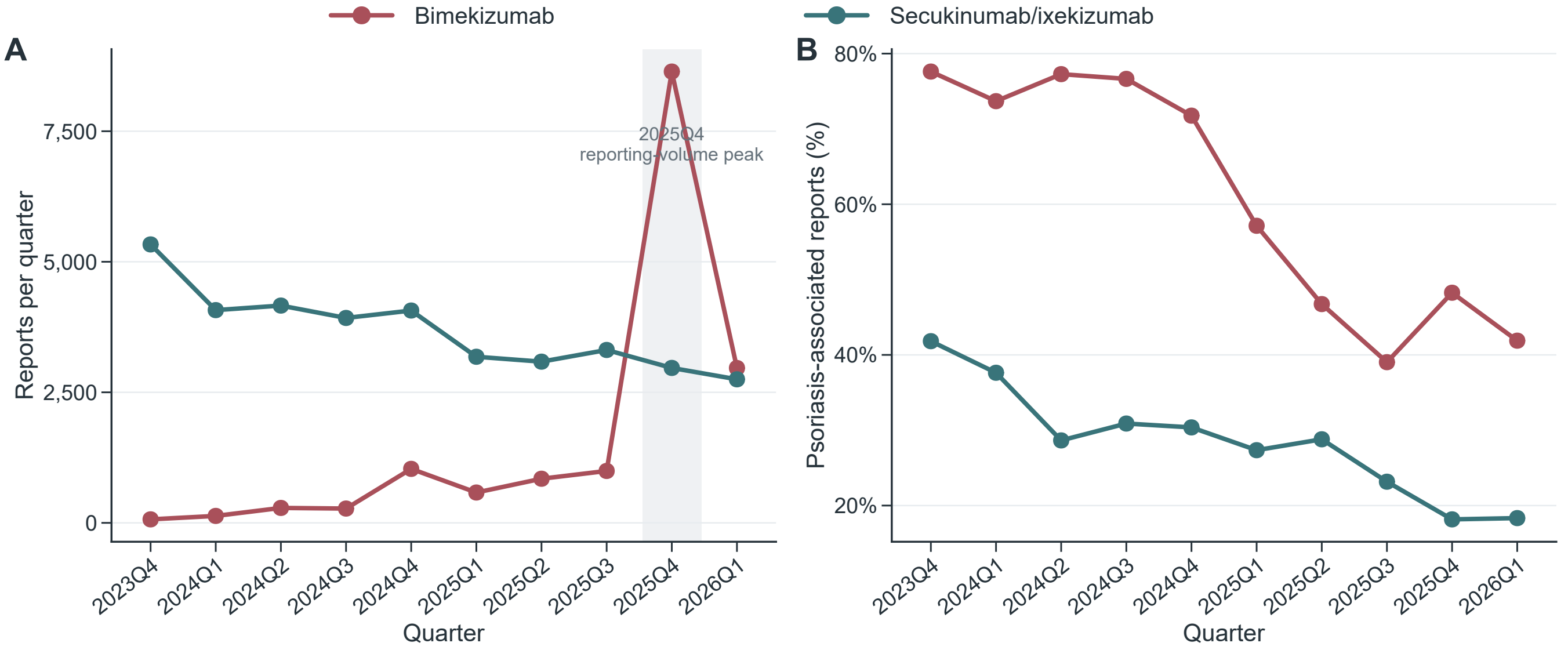

Bimekizumab
Secukinumab/ixekizumab
A
Reports per quarter
7,500
5,000
2,500
0
2025Q4
reporting-volume peak
2023Q4
2024Q1
2024Q2
2024Q3
2024Q4
2025Q1
2025Q2
2025Q3
2025Q4
2026Q1
Quarter
B
Psoriasis-associated reports (%)
80%
60%
40%
20%
Quarter

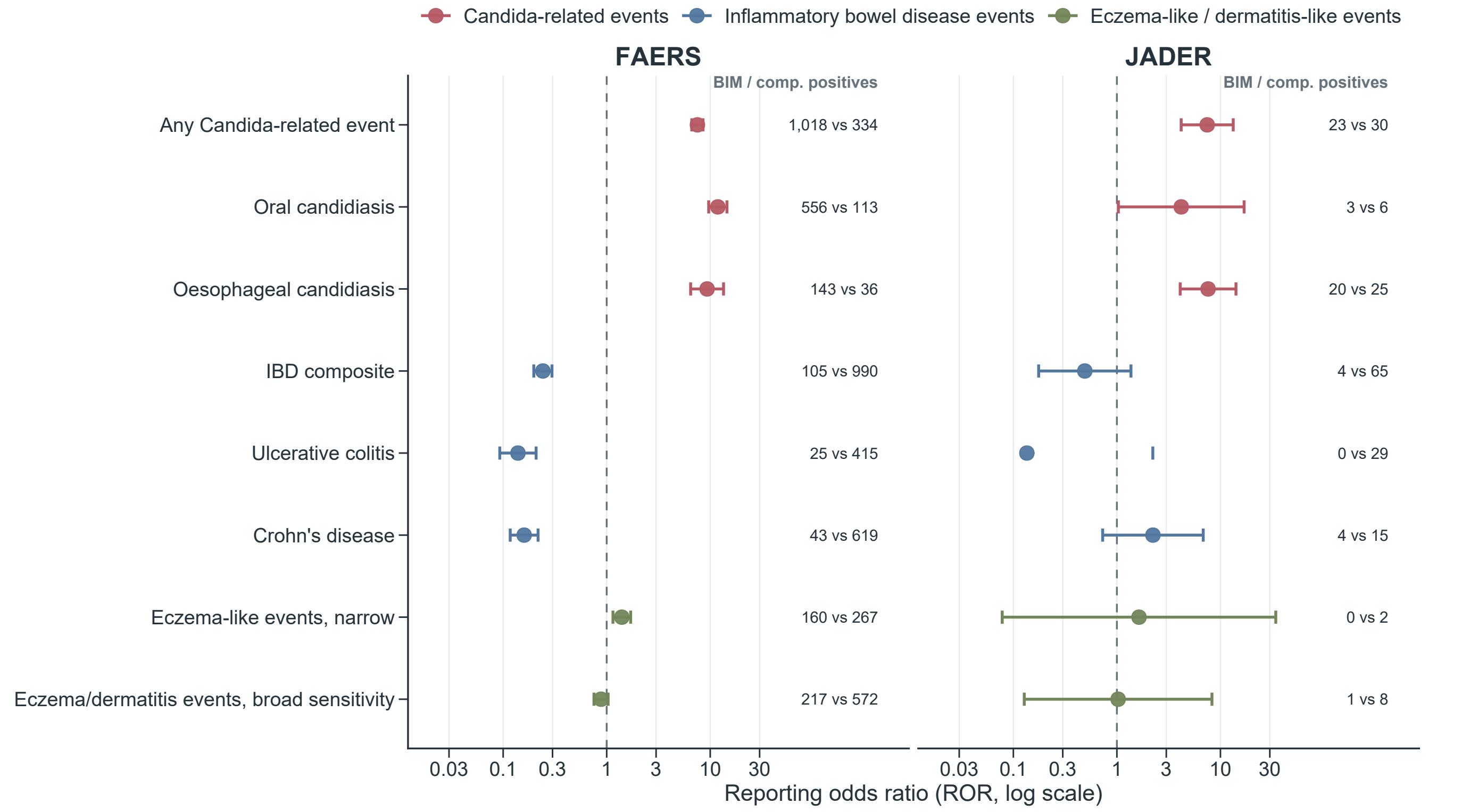
Candida-related events
Inflammatory bowel disease events
Eczema-like / dermatitis-like events
FAERS
JADER
BIM / comp. positives
Any Candida-related event
Oral candidiasis
Oesophageal candidiasis
IBD composite
Ulcerative colitis
Crohn's disease
Eczema-like events, narrow
Eczema/dermatitis events, broad sensitivity
1,018 vs 334
556 vs 113
143 vs 36
105 vs 990
25 vs 415
43 vs 619
160 vs 267
217 vs 572
23 vs 30
3 vs 6
20 vs 25
4 vs 65
0 vs 29
4 vs 15
0 vs 2
1 vs 8
0.03
0.1
0.3
1
3
10
30
Reporting odds ratio (ROR, log scale)

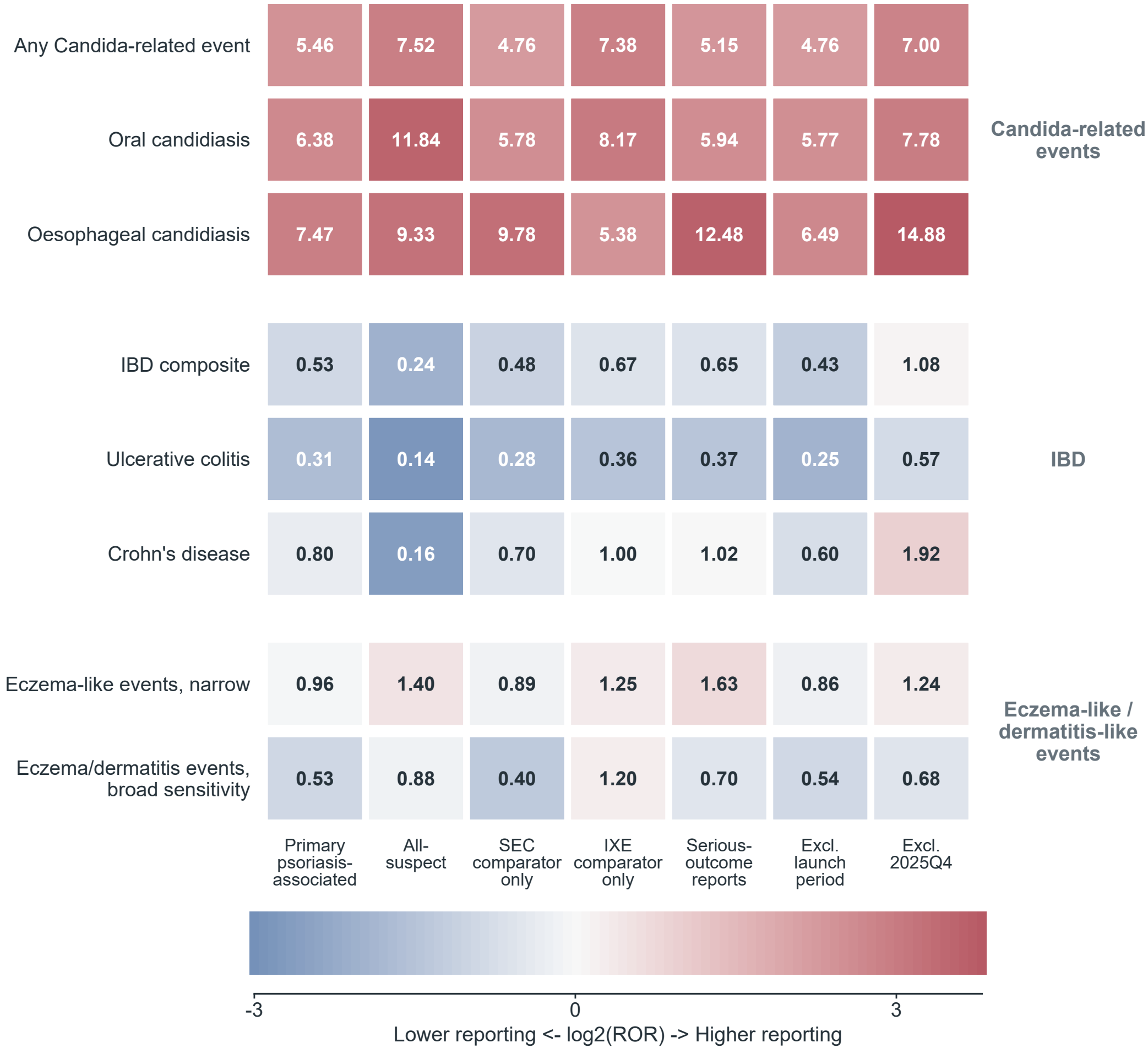

Any Candida-related event
5.46
7.52
4.76
7.38
5.15
4.76
7.00
Oral candidiasis
6.38
11.84
5.78
8.17
5.94
5.77
7.78
Oesophageal candidiasis
7.47
9.33
9.78
5.38
12.48
6.49
14.88
Candida-related events
IBD composite
0.53
0.24
0.48
0.67
0.65
0.43
1.08
Ulcerative colitis
0.31
0.14
0.28
0.36
0.37
0.25
0.57
Crohn's disease
0.80
0.16
0.70
1.00
1.02
0.60
1.92
IBD
Eczema-like events, narrow
0.96
1.40
0.89
1.25
1.63
0.86
1.24
Eczema/dermatitis events, broad sensitivity
0.53
0.88
0.40
1.20
0.70
0.54
0.68
Eczema-like / dermatitis-like events
Primary psoriasis-associated
All-suspect
SEC comparator only
IXE comparator only
Serious-outcome reports
Excl. launch period
Excl. 2025Q4
-3
0
3
Lower reporting <- log2(ROR) -> Higher reporting

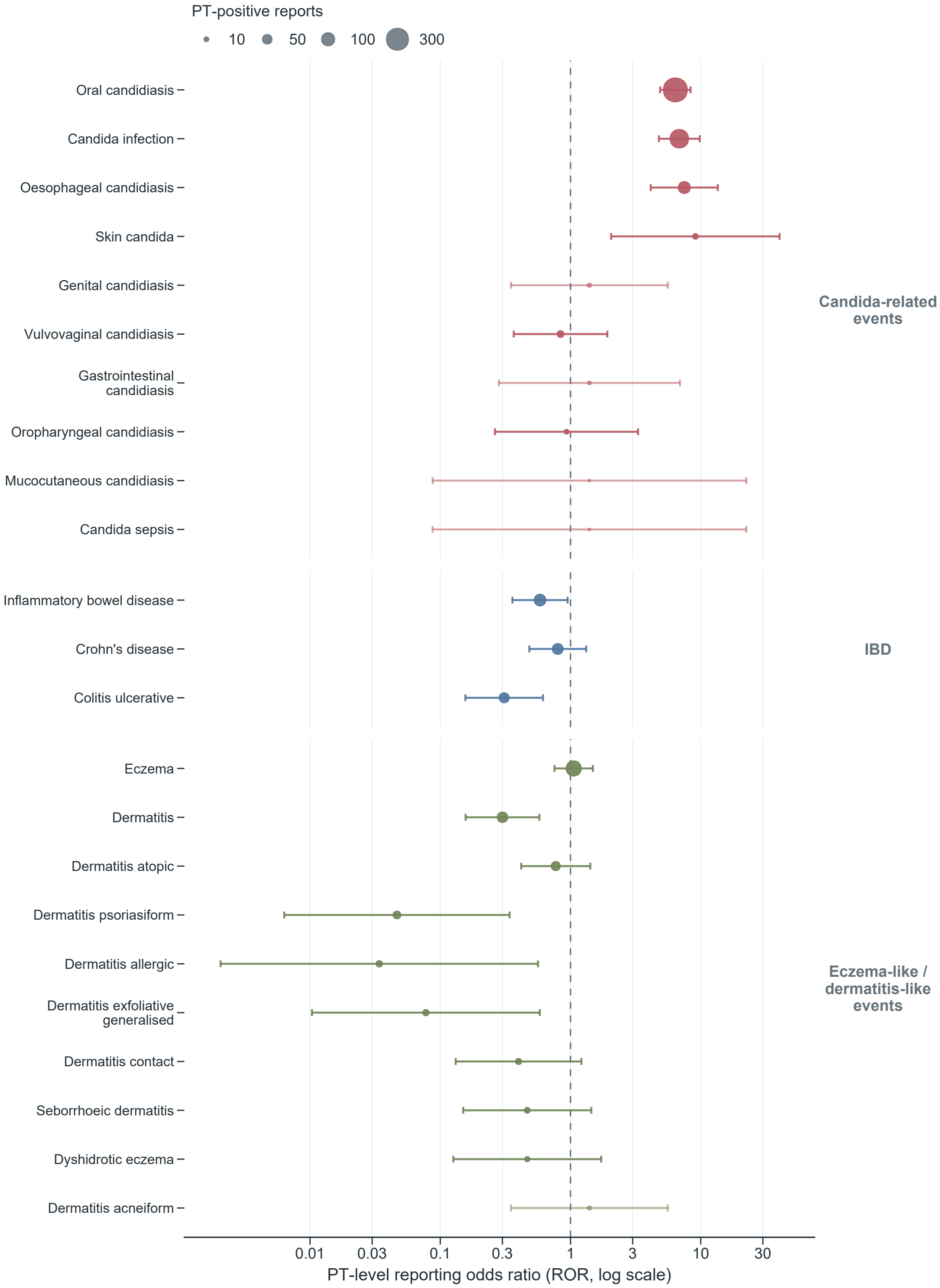

PT-positive reports
10
50
100
300
Oral candidiasis
Candida infection
Oesophageal candidiasis
Skin candida
Genital candidiasis
Vulvovaginal candidiasis
Gastrointestinal candidiasis
Oropharyngeal candidiasis
Mucocutaneous candidiasis
Candida sepsis
Candida-related events
Inflammatory bowel disease
Crohn's disease
Colitis ulcerative
IBD
Eczema
Dermatitis
Dermatitis atopic
Dermatitis psoriasiform
Dermatitis allergic
Dermatitis exfoliative generalised
Dermatitis contact
Seborrhoeic dermatitis
Dyshidrotic eczema
Dermatitis acneiform
Eczema-like / dermatitis-like events
0.01
0.03
0.1
0.3
1
3
10
30
PT-level reporting odds ratio (ROR, log scale)

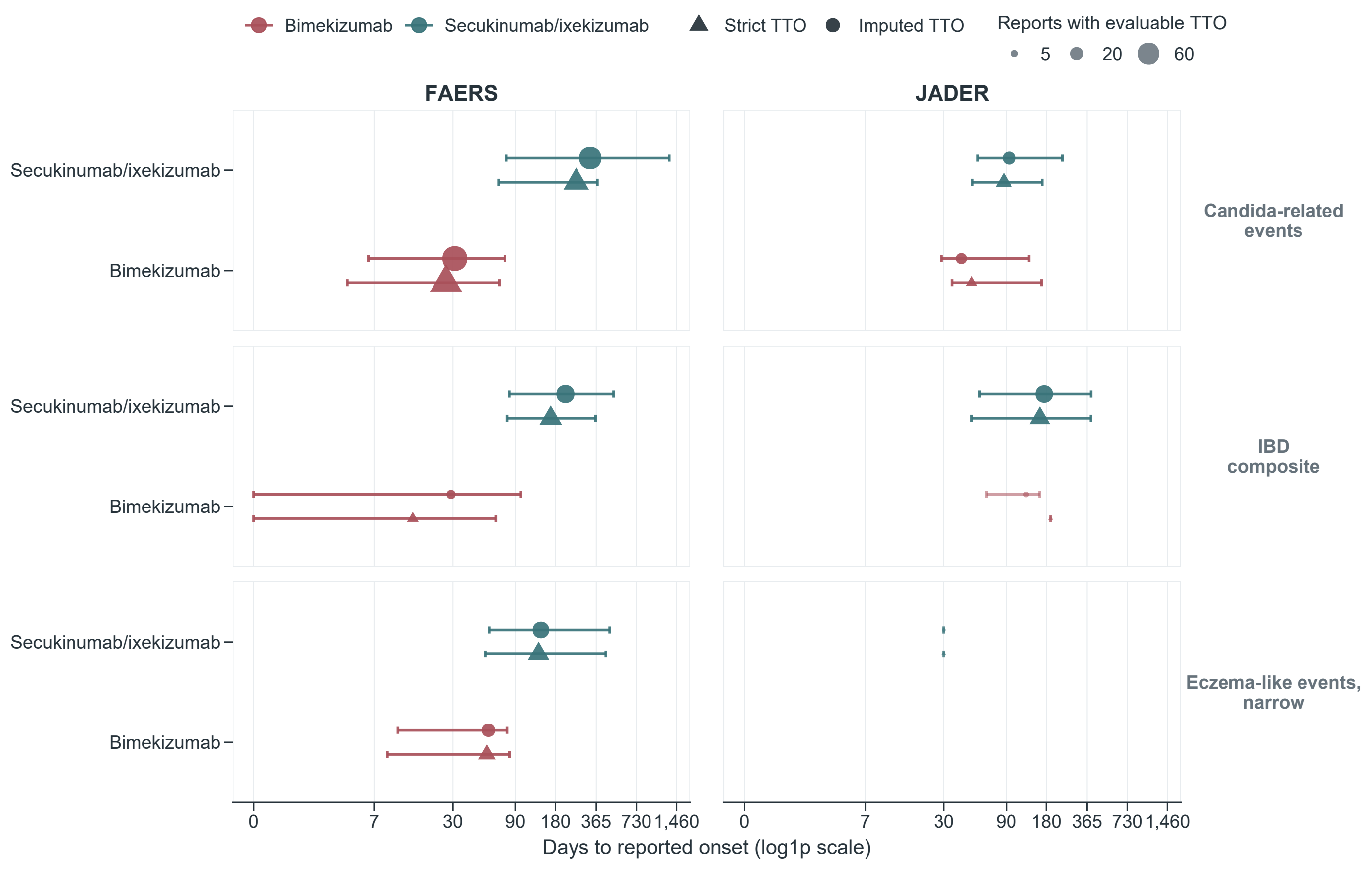

Bimekizumab
Secukinumab/ixekizumab
Strict TTO
Imputed TTO
Reports with evaluable TTO
5
20
60
FAERS
JADER
Secukinumab/ixekizumab
Bimekizumab
Candida-related events
IBD composite
Eczema-like events, narrow
0
7
30
90
180
365
730
1,460
Days to reported onset (log1p scale)

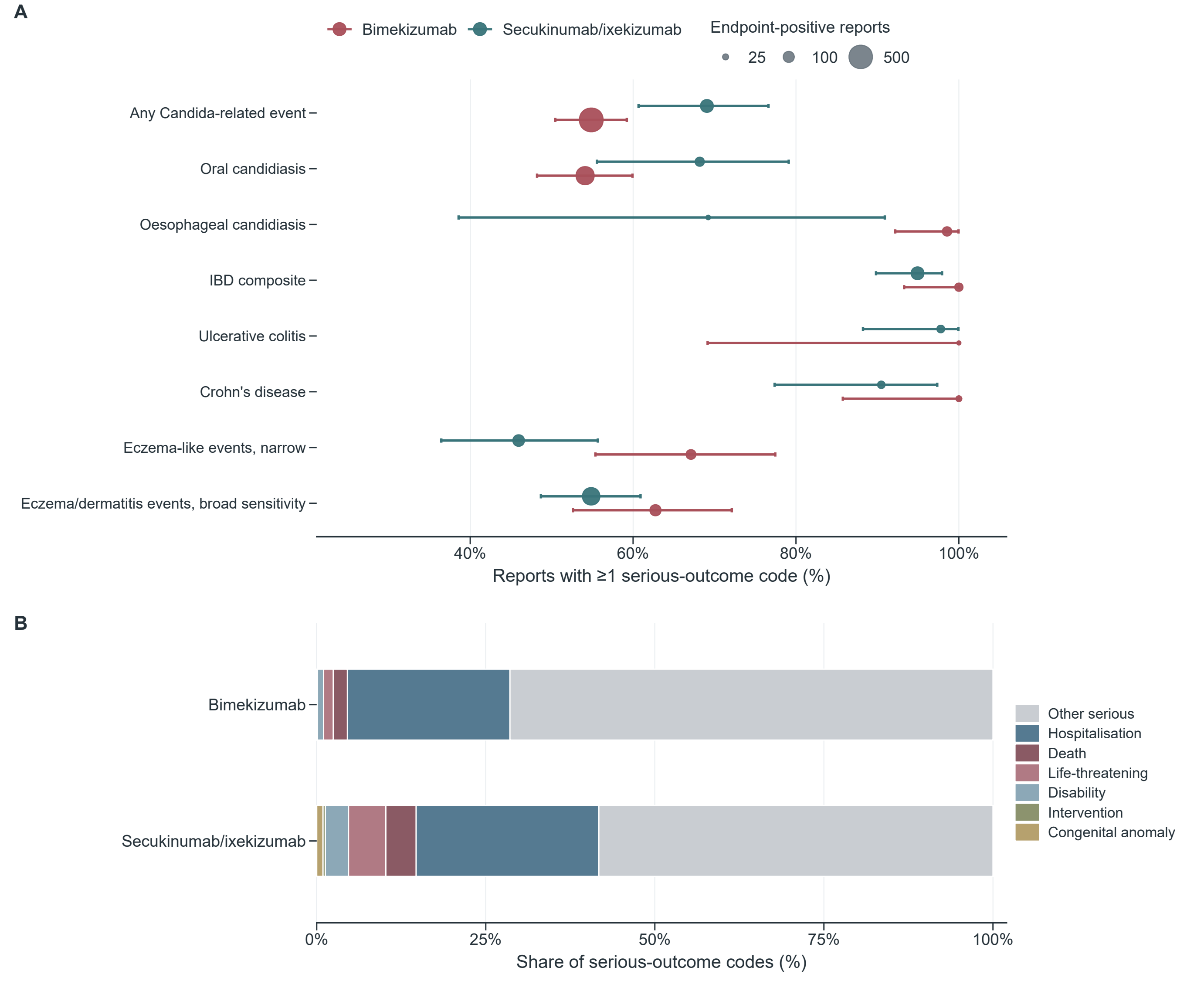

A
Bimekizumab
Secukinumab/ixekizumab
Endpoint-positive reports
25
100
500
Any Candida-related event
Oral candidiasis
Oesophageal candidiasis
IBD composite
Ulcerative colitis
Crohn's disease
Eczema-like events, narrow
Eczema/dermatitis events, broad sensitivity
40%
60%
80%
100%
Reports with ≥1 serious-outcome code (%)
B
Bimekizumab
Secukinumab/ixekizumab
Other serious
Hospitalisation
Death
Life-threatening
Disability
Intervention
Congenital anomaly
0%
25%
50%
75%
100%
Share of serious-outcome codes (%)

Supplementary Table S1. Study design and analysis specifications.

Component	Specification
FAERS data window	2023 Q4 through 2026 Q1
JADER release	2026-05
FAERS deduplication	caseid; retain highest caseversion and highest primaryid as tie-breaker
JADER case key	identifier with report count
Exposure role	FAERS primary or secondary suspect; JADER suspected drug
Target drugs	bimekizumab, secukinumab, and ixekizumab, including brand names and Japanese aliases
Main indication	psoriasis-associated reports/cases defined using prespecified psoriasis and psoriatic-disease indication terms
Primary comparator	pooled secukinumab/ixekizumab
Counting unit	FAERS deduplicated reports; JADER reconstructed case-level reports with a prespecified endpoint-positive flag
Primary estimands	ROR as the prespecified primary disproportionality metric; PRR and IC as supportive pharmacovigilance metrics in complete report-level and PT-level supplementary analyses
TTO primary rule	complete start and event dates; non-negative values
Continuity correction	A 0.5 continuity correction was applied when any cell in a 2 x 2 table was zero.
Sensitivity analyses	All-suspect, SEC-only, IXE-only, serious-outcome reports, launch-period exclusion, and 2025Q4 exclusion analyses were used as supplementary robustness checks.
Interpretation constraints	Disproportionality estimates describe reporting enrichment and do not estimate incidence, causality, or comparative clinical risk.

Abbreviations: FAERS, FDA Adverse Event Reporting System; IC, information component; JADER, Japanese Adverse Drug Event Report database; PRR, proportional reporting ratio; ROR, reporting odds ratio; TTO, time to onset.

Supplementary Table S2. Prespecified adverse-event endpoint dictionary.

Domain	Endpoint	Endpoint key	Operational definition
Candida-related events	Any Candida-related event	any_candidiasis	Any MedDRA PT containing candid-; interpreted as a Candida-related endpoint rather than a strictly mucocutaneous endpoint
	Oral candidiasis	oral_candidiasis	Oral candidiasis
	Oesophageal candidiasis	oesophageal_candidiasis	Oesophageal or esophageal candidiasis
Inflammatory bowel disease events	IBD composite	ibd_composite	Inflammatory bowel disease, ulcerative colitis, or Crohn disease terms
	Ulcerative colitis	colitis_ulcerative	Colitis ulcerative or ulcerative colitis terms
	Crohn's disease	crohns_disease	Crohn's disease terms
Eczema-like / dermatitis-like events	Eczema-like events, narrow	eczema_narrow	Eczema, atopic dermatitis, nummular eczema, dyshidrotic eczema, pompholyx, or rash eczematous
	Eczema/dermatitis events, broad sensitivity	eczema_broad	Narrow definition plus any PT containing eczema or dermatitis; used as a broad sensitivity endpoint

Operational definitions use MedDRA preferred-term text searches or prespecified term sets. The broad eczema/dermatitis endpoint was used as a sensitivity endpoint. The term "narrow" denotes a more specific author-defined PT set rather than a formal Standardised MedDRA Query (SMQ) narrow search.

Supplementary Table S3. Complete report-level disproportionality results.

Source	Scope	Safety domain	Endpoint	Bimekizumab n/N	Comparator n/N	ROR (95% CI)	PRR (95% CI)	IC (IC025-IC975)	Chi-square	ROR signal	PRR signal	IC signal	0.5 correction
FAERS	All-suspect	Candida-related events	Any Candida-related event	1,018/15,821	334/36,852	7.52 (6.64-8.52)	7.10 (6.28-8.02)	1.33 (1.16-1.49)	1352.61	Yes	Yes	Yes	No
			Oral candidiasis	556/15,821	113/36,852	11.84 (9.67-14.51)	11.46 (9.37-14.02)	1.47 (1.24-1.70)	908.25	Yes	Yes	Yes	No
			Oesophageal candidiasis	143/15,821	36/36,852	9.33 (6.47-13.45)	9.25 (6.42-13.33)	1.41 (0.96-1.86)	212.41	Yes	Yes	Yes	No
		Inflammatory bowel disease events	IBD composite	105/15,821	990/36,852	0.24 (0.20-0.30)	0.25 (0.20-0.30)	-1.65 (-1.96--1.33)	222.48	No	No	No	No
			Ulcerative colitis	25/15,821	415/36,852	0.14 (0.09-0.21)	0.14 (0.09-0.21)	-2.40 (-3.02--1.79)	125.24	No	No	No	No
			Crohn's disease	43/15,821	619/36,852	0.16 (0.12-0.22)	0.16 (0.12-0.22)	-2.21 (-2.68--1.73)	176.80	No	No	No	No
		Eczema-like / dermatitis-like events	Eczema-like events, narrow	160/15,821	267/36,852	1.40 (1.15-1.70)	1.40 (1.15-1.70)	0.32 (-0.02-0.65)	11.32	Yes	No	No	No
			Eczema/dermatitis events, broad sensitivity	217/15,821	572/36,852	0.88 (0.75-1.03)	0.88 (0.76-1.03)	-0.13 (-0.39-0.14)	2.45	No	No	No	No
	Psoriasis-associated	Candida-related events	Any Candida-related event	514/7,850	139/10,971	5.46 (4.52-6.60)	5.17 (4.29-6.22)	0.92 (0.70-1.13)	381.01	Yes	Yes	Yes	No
			Oral candidiasis	292/7,850	66/10,971	6.38 (4.88-8.35)	6.18 (4.74-8.06)	0.97 (0.68-1.25)	238.43	Yes	Yes	Yes	No
			Oesophageal candidiasis	69/7,850	13/10,971	7.47 (4.13-13.53)	7.42 (4.10-13.41)	1.01 (0.42-1.60)	61.01	Yes	Yes	Yes	No
		Inflammatory bowel disease events	IBD composite	53/7,850	138/10,971	0.53 (0.39-0.73)	0.54 (0.39-0.74)	-0.59 (-1.09--0.09)	15.47	No	No	No	No
			Ulcerative colitis	10/7,850	45/10,971	0.31 (0.16-0.61)	0.31 (0.16-0.62)	-1.20 (-2.27--0.13)	12.56	No	No	No	No
			Crohn's disease	24/7,850	42/10,971	0.80 (0.48-1.32)	0.80 (0.48-1.32)	-0.20 (-0.99-0.59)	0.78	No	No	No	No
		Eczema-like / dermatitis-like events	Eczema-like events, narrow	76/7,850	111/10,971	0.96 (0.71-1.28)	0.96 (0.72-1.28)	-0.04 (-0.49-0.42)	0.09	No	No	No	No
			Eczema/dermatitis events, broad sensitivity	102/7,850	268/10,971	0.53 (0.42-0.66)	0.53 (0.42-0.67)	-0.60 (-0.96--0.24)	31.04	No	No	No	No

Source	Scope	Safety domain	Endpoint	Bimekizumab n/N	Comparator n/N	ROR (95% CI)	PRR (95% CI)	IC (IC025-IC975)	Chi-square	ROR signal	PRR signal	IC signal	0.5 correction
JADER	All-suspect	Candida-related events	Any Candida-related event	23/129	30/1,060	7.45 (4.18-13.29)	6.30 (3.78-10.51)	2.00 (0.68-3.32)	60.75	Yes	Yes	Yes	No
			Oral candidiasis	3/129	6/1,060	4.18 (1.03-16.93)	4.11 (1.04-16.23)	1.62 (-1.68-4.91)	4.74	Yes	Yes	No	No
			Oesophageal candidiasis	20/129	25/1,060	7.60 (4.09-14.12)	6.57 (3.76-11.50)	2.03 (0.61-3.46)	54.57	Yes	Yes	Yes	No
		Inflammatory bowel disease events	IBD composite	4/129	65/1,060	0.49 (0.18-1.37)	0.51 (0.19-1.36)	-0.90 (-2.66-0.85)	1.93	No	No	No	No
			Ulcerative colitis	0/129	29/1,060	0.14 (0.01-2.22)	0.14 (0.01-2.25)	-2.71 (-7.00-1.58)	2.71	No	No	No	Yes
			Crohn's disease	4/129	15/1,060	2.23 (0.73-6.82)	2.19 (0.74-6.50)	0.96 (-1.47-3.38)	2.08	No	No	No	No
		Eczema-like / dermatitis-like events	Eczema-like events, narrow	0/129	2/1,060	1.63 (0.08-34.24)	1.63 (0.08-33.82)	0.61 (-5.75-6.97)	0.10	No	No	No	Yes
			Eczema/dermatitis events, broad sensitivity	1/129	8/1,060	1.03 (0.13-8.28)	1.03 (0.13-8.15)	0.03 (-3.99-4.06)	0.00	No	No	No	No
	Psoriasis-associated	Candida-related events	Any Candida-related event	19/104	25/890	7.73 (4.09-14.62)	6.50 (3.71-11.40)	2.05 (0.58-3.51)	52.61	Yes	Yes	Yes	No
			Oral candidiasis	2/104	5/890	3.47 (0.66-18.12)	3.42 (0.67-17.42)	1.45 (-2.41-5.31)	2.47	No	No	No	No
			Oesophageal candidiasis	17/104	20/890	8.50 (4.29-16.83)	7.27 (3.94-13.44)	2.13 (0.54-3.73)	51.65	Yes	Yes	Yes	No
		Inflammatory bowel disease events	IBD composite	3/104	55/890	0.45 (0.14-1.47)	0.47 (0.15-1.47)	-1.02 (-3.01-0.98)	1.84	No	No	No	No
			Ulcerative colitis	0/104	27/890	0.15 (0.01-2.48)	0.15 (0.01-2.51)	-2.56 (-6.89-1.76)	2.34	No	No	No	Yes
			Crohn's disease	3/104	10/890	2.61 (0.71-9.65)	2.57 (0.72-9.18)	1.14 (-1.78-4.06)	2.24	No	No	No	No
		Eczema-like / dermatitis-like events	Eczema-like events, narrow	0/104	2/890	1.70 (0.08-35.66)	1.70 (0.08-35.11)	0.66 (-5.76-7.09)	0.12	No	No	No	Yes
			Eczema/dermatitis events, broad sensitivity	0/104	8/890	0.50 (0.03-8.67)	0.50 (0.03-8.59)	-0.92 (-5.87-4.02)	0.24	No	No	No	Yes

ROR was the prespecified primary disproportionality metric. PRR and IC were calculated as supportive pharmacovigilance metrics. A 0.5 continuity correction was applied when any cell in a 2 x 2 table was zero.

Supplementary Table S4. Data dictionary for the machine-readable PT-level supportive disproportionality output.

Column in Supplementary Data 1	Description
source	Database source used for the PT-level supportive analysis.
scope	Analysis scope, including all-suspect or psoriasis-associated analyses.
pt	MedDRA Preferred Term evaluated in the PT-level output.
endpoint tags	Prespecified safety-domain tag(s), where applicable; NA indicates that the PT was not assigned to a prespecified safety-domain endpoint.
bimekizumab total	Number of bimekizumab reports/cases in the corresponding analysis stratum.
bimekizumab positive	Number of bimekizumab reports/cases containing the PT.
comparator total	Number of pooled secukinumab/ixekizumab comparator reports/cases in the corresponding analysis stratum.
comparator positive	Number of pooled secukinumab/ixekizumab comparator reports/cases containing the PT.
total positive	Total PT-positive reports/cases across bimekizumab and pooled comparator strata.
ror	Reporting odds ratio for bimekizumab versus pooled secukinumab/ixekizumab.
ror lcl	Lower bound of the 95% confidence interval for the ROR.
ror ucl	Upper bound of the 95% confidence interval for the ROR.
prr	Proportional reporting ratio, calculated as a supportive pharmacovigilance metric.
prr lcl	Lower bound of the 95% confidence interval for the PRR.
prr ucl	Upper bound of the 95% confidence interval for the PRR.
ic	Information component, calculated as a supportive pharmacovigilance metric.
ic025	Lower credibility bound for the information component.
ic975	Upper credibility bound for the information component.
chi square	Chi-square statistic from the corresponding 2 x 2 table.
ror signal	Indicator for whether the PT-level ROR signal rule was met.
prr signal	Indicator for whether the PT-level PRR signal rule was met.
ic signal	Indicator for whether the PT-level IC signal rule was met.
continuity corrected	Indicator for whether a 0.5 continuity correction was applied because at least one 2 x 2 table cell was zero.

The complete PT-level output contains 9,974 rows and 23 columns and is provided as Supplementary Data 1 rather than as a full Word table. PT, Preferred Term; ROR, reporting odds ratio; PRR, proportional reporting ratio; IC, information component.

Supplementary Table S5. FAERS ROR sensitivity analyses.

Analysis	Safety domain	Endpoint	Bimekizumab n/N	Comparator n/N	ROR (95% CI)	0.5 correction
Primary psoriasis-associated	Candida-related events	Any Candida-related event	514/7,850	139/10,971	5.46 (4.52-6.60)	No
		Oral candidiasis	292/7,850	66/10,971	6.38 (4.88-8.35)	No
		Oesophageal candidiasis	69/7,850	13/10,971	7.47 (4.13-13.53)	No
	Inflammatory bowel disease events	IBD composite	53/7,850	138/10,971	0.53 (0.39-0.73)	No
		Ulcerative colitis	10/7,850	45/10,971	0.31 (0.16-0.61)	No
		Crohn's disease	24/7,850	42/10,971	0.80 (0.48-1.32)	No
	Eczema-like / dermatitis-like events	Eczema-like events, narrow	76/7,850	111/10,971	0.96 (0.71-1.28)	No
		Eczema/dermatitis events, broad sensitivity	102/7,850	268/10,971	0.53 (0.42-0.66)	No
All-suspect	Candida-related events	Any Candida-related event	1,018/15,821	334/36,852	7.52 (6.64-8.52)	No
		Oral candidiasis	556/15,821	113/36,852	11.84 (9.67-14.51)	No
		Oesophageal candidiasis	143/15,821	36/36,852	9.33 (6.47-13.45)	No
	Inflammatory bowel disease events	IBD composite	105/15,821	990/36,852	0.24 (0.20-0.30)	No
		Ulcerative colitis	25/15,821	415/36,852	0.14 (0.09-0.21)	No
		Crohn's disease	43/15,821	619/36,852	0.16 (0.12-0.22)	No
	Eczema-like / dermatitis-like events	Eczema-like events, narrow	160/15,821	267/36,852	1.40 (1.15-1.70)	No
		Eczema/dermatitis events, broad sensitivity	217/15,821	572/36,852	0.88 (0.75-1.03)	No
SEC comparator only	Candida-related events	Any Candida-related event	514/7,850	96/6,623	4.76 (3.82-5.94)	No
		Oral candidiasis	292/7,850	44/6,623	5.78 (4.20-7.94)	No
		Oesophageal candidiasis	69/7,850	6/6,623	9.78 (4.24-22.54)	No
	Inflammatory bowel disease events	IBD composite	53/7,850	93/6,623	0.48 (0.34-0.67)	No
		Ulcerative colitis	10/7,850	30/6,623	0.28 (0.14-0.57)	No
		Crohn's disease	24/7,850	29/6,623	0.70 (0.41-1.20)	No
	Eczema-like / dermatitis-like events	Eczema-like events, narrow	76/7,850	72/6,623	0.89 (0.64-1.23)	No
		Eczema/dermatitis events, broad sensitivity	102/7,850	210/6,623	0.40 (0.32-0.51)	No
IXE comparator only	Candida-related events	Any Candida-related event	514/7,850	40/4,251	7.38 (5.34-10.20)	No
		Oral candidiasis	292/7,850	20/4,251	8.17 (5.19-12.88)	No
		Oesophageal candidiasis	69/7,850	7/4,251	5.38 (2.47-11.71)	No

Analysis	Safety domain	Endpoint	Bimekizumab n/N	Comparator n/N	ROR (95% CI)	0.5 correction
	Inflammatory bowel disease events	IBD composite	53/7,850	43/4,251	0.67 (0.44-1.00)	No
		Ulcerative colitis	10/7,850	15/4,251	0.36 (0.16-0.80)	No
		Crohn's disease	24/7,850	13/4,251	1.00 (0.51-1.97)	No
	Eczema-like / dermatitis-like events	Eczema-like events, narrow	76/7,850	33/4,251	1.25 (0.83-1.88)	No
		Eczema/dermatitis events, broad sensitivity	102/7,850	46/4,251	1.20 (0.85-1.71)	No
Serious-outcome reports	Candida-related events	Any Candida-related event	282/2,916	96/4,714	5.15 (4.07-6.52)	No
		Oral candidiasis	158/2,916	45/4,714	5.94 (4.25-8.31)	No
		Oesophageal candidiasis	68/2,916	9/4,714	12.48 (6.22-25.05)	No
	Inflammatory bowel disease events	IBD composite	53/2,916	131/4,714	0.65 (0.47-0.89)	No
		Ulcerative colitis	10/2,916	44/4,714	0.37 (0.18-0.73)	No
		Crohn's disease	24/2,916	38/4,714	1.02 (0.61-1.71)	No
	Eczema-like / dermatitis-like events	Eczema-like events, narrow	51/2,916	51/4,714	1.63 (1.10-2.41)	No
		Eczema/dermatitis events, broad sensitivity	64/2,916	147/4,714	0.70 (0.52-0.94)	No
Excl. launch period	Candida-related events	Any Candida-related event	492/7,700	102/7,208	4.76 (3.83-5.90)	No
		Oral candidiasis	275/7,700	46/7,208	5.77 (4.21-7.89)	No
		Oesophageal candidiasis	62/7,700	9/7,208	6.49 (3.22-13.07)	No
	Inflammatory bowel disease events	IBD composite	50/7,700	109/7,208	0.43 (0.30-0.60)	No
		Ulcerative colitis	9/7,700	33/7,208	0.25 (0.12-0.53)	No
		Crohn's disease	23/7,700	36/7,208	0.60 (0.35-1.01)	No
	Eczema-like / dermatitis-like events	Eczema-like events, narrow	70/7,700	76/7,208	0.86 (0.62-1.19)	No
		Eczema/dermatitis events, broad sensitivity	95/7,700	164/7,208	0.54 (0.42-0.69)	No
Excl. 2025Q4	Candida-related events	Any Candida-related event	288/3,680	125/10,432	7.00 (5.66-8.67)	No
		Oral candidiasis	156/3,680	59/10,432	7.78 (5.75-10.53)	No
		Oesophageal candidiasis	62/3,680	12/10,432	14.88 (8.01-27.64)	No
	Inflammatory bowel disease events	IBD composite	48/3,680	126/10,432	1.08 (0.77-1.51)	No
		Ulcerative colitis	8/3,680	40/10,432	0.57 (0.26-1.21)	No
		Crohn's disease	23/3,680	34/10,432	1.92 (1.13-3.27)	No
	Eczema-like / dermatitis-like events	Eczema-like events, narrow	46/3,680	105/10,432	1.24 (0.88-1.76)	No

Analysis	Safety domain	Endpoint	Bimekizumab n/N	Comparator n/N	ROR (95% CI)	0.5 correction
		Eczema/dermatitis events, broad sensitivity	60/3,680	249/10,432	0.68 (0.51-0.90)	No

Complete cell counts and ROR estimates are shown for each prespecified sensitivity analysis. The 2025Q4 exclusion analysis was used as a reporting-volume stress test.

Supplementary Table S6. Reported time-to-onset summaries under strict and imputed onset definitions.

Source	Group	Endpoint	Endpoint-positive records in the TTO source dataset	Strict evaluable TTO, n	Strict TTO median (IQR), days	Imputed evaluable TTO, n	Imputed TTO median (IQR), days
FAERS	Bimekizumab	Any Candida-related event	182	74	26.5 (4-67.8)	80	31 (6.2-74.8)
		Ulcerative colitis	4	2	14.5 (7.2-21.8)	2	14.5 (7.2-21.8)
		Crohn's disease	14	5	0 (0-52)	6	26 (0-108.2)
		Eczema/dermatitis events, broad sensitivity	40	22	50 (11-73.5)	23	53 (11-72.5)
		Eczema-like events, narrow	32	20	54.5 (9-81.5)	21	56 (11-78)
		IBD composite	22	8	14.5 (0-63.8)	9	29 (0-99)
		Oesophageal candidiasis	33	23	31 (15.5-69.5)	23	31 (15.5-69.5)
		Oral candidiasis	108	41	17 (4-58)	46	24.5 (7-67.8)
	Secukinumab/ixekizumab	Any Candida-related event	98	45	258 (67-372)	64	329 (76.8-1286)
		Ulcerative colitis	31	21	186 (94-300)	23	214 (94-439.5)
		Crohn's disease	24	5	139 (5-362)	9	139 (58-685)
		Eczema/dermatitis events, broad sensitivity	142	77	138 (44-615)	89	163 (46-700)
		Eczema-like events, narrow	60	32	134.5 (53-430.8)	34	140 (56.8-460.8)
		IBD composite	68	34	166 (78-361.2)	41	214 (81-493)
		Oesophageal candidiasis	4	2	49.5 (28.8-70.2)	3	17 (12.5-54)
		Oral candidiasis	52	21	372 (84-372)	37	372 (195-1738)
JADER	Bimekizumab	Any Candida-related event	14	8	49 (34.8-166)	14	41 (28.8-133.5)
		Crohn's disease	3	1	194 (194-194)	3	127 (63.5-160.5)
		IBD composite	3	1	194 (194-194)	3	127 (63.5-160.5)
		Oesophageal candidiasis	12	7	61 (34-178)	12	41 (25.8-92.5)
		Oral candidiasis	2	1	36 (36-36)	2	343 (189.5-496.5)
	Secukinumab/ixekizumab	Any Candida-related event	23	18	86 (49.5-167.5)	20	94.5 (54.5-238)
		Ulcerative colitis	25	15	222 (56.5-390.5)	22	224.5 (94-390.8)
		Crohn's disease	10	5	161 (44-186)	6	173.5 (73.2-372)
		Eczema/dermatitis events, broad sensitivity	5	1	30 (30-30)	4	20.5 (8.2-33.5)
		Eczema-like events, narrow	2	1	30 (30-30)	1	30 (30-30)
		IBD composite	53	29	161 (49-390)	38	173.5 (56.2-390.8)
		Oesophageal candidiasis	18	15	81 (52-196)	17	91 (57-265)
		Oral candidiasis	5	5	57 (28-98)	5	57 (28-98)

Note: Endpoint-positive counts refer to records included in the TTO source dataset, not all endpoint-positive reports/cases in the primary analysis. Strict TTO required complete non-negative start and onset dates; imputed TTO used prespecified partial-date imputation. Results are descriptive only. IQR, interquartile range; TTO, time to onset.

Supplementary Table S7. Label, pooled-trial, and case-literature contextual evidence.

Drug	Safety domain	Evidence stream	Source or study	Population or case	Exposure or onset	Key finding	Clinical interpretation	Citation or search record	Evidence level
Bimekizumab	Candida-related events	FDA prescribing information	BIMZELX USPI, Sections 5.2 and 6.1	Adult plaque psoriasis, Trials Ps-1 and Ps-2; BIMZELX n=670, placebo n=169	Placebo-controlled through week 16	Oral candidiasis occurred in 61/670 (9%) vs 0/169 placebo; other Candida infections occurred in 7/670 (1%) vs 1/169 (1%).	Oral candidiasis is a common labeled adverse reaction and is usually mucocutaneous; infection monitoring is required.	BIMZELX (bimekizumab-bkzx) US Prescribing Information. UCB; revised 06/2025; FDA SPL set ID 26b88358-871f-4c80-9d80-b2fb16477f81, version 14.	Regulatory label
		Clinical / pooled evidence	BE VIVID, BE READY, BE SURE, and BE BRIGHT pooled 3-year safety	1,495 bimekizumab-treated patients; 3,876.4 patient-years	Up to 3 years	Oral candidiasis EAIR was 10.1/100 patient-years; 99.3% of events were mild or moderate, none was serious, and few led to discontinuation.	The increased signal is clinically coherent and generally manageable, but recurrence and esophageal disease remain relevant surveillance issues.	Bimekizumab safety in plaque psoriasis: pooled data through 3 years. Br J Dermatol. 2024. doi:10.1093/bjd/ljad429; PMID:37950894.	Pooled phase III and extension
		Case-based literature	Targeted PubMed case-report search	Bimekizumab-treated psoriasis patients	Search through 2026-06-18	No eligible single-patient candidiasis report involving bimekizumab for psoriasis was identified.	This is a negative search result, not evidence that clinically important candidiasis does not occur; trial and label evidence remain stronger.	PubMed queries: bimekizumab AND candidiasis AND case report; bimekizumab AND esophageal/oral candidiasis. Search date 2026-06-18. A systemic-sclerosis case with mild oral candidiasis (PMID:41884839) was excluded because the indication was not psoriasis.	Negative literature search
	Inflammatory bowel disease	FDA prescribing information	BIMZELX USPI, Section 5.5 and plaque psoriasis safety	2,480 exposed plaque psoriasis patients; 5,830 patient-years	Clinical development program	Seven adjudicated new-onset IBD cases occurred (0.12/100 patient-years); most were serious and led to discontinuation. Active IBD should be avoided and new or worsening IBD requires discontinuation.	Absolute frequency was low, but the labeled warning supports active screening for gastrointestinal symptoms and prior IBD.	BIMZELX (bimekizumab-bkzx) US Prescribing Information. UCB; revised 06/2025; FDA SPL set ID 26b88358-871f-4c80-9d80-b2fb16477f81, version 14.	Regulatory label
		Clinical /	BE VIVID,	1,495 patients;	Up to 3 years	Adjudicated IBD EAIR	The pooled rate	Bimekizumab safety in plaque	Pooled phase

Drug	Safety domain	Evidence stream	Source or study	Population or case	Exposure or onset	Key finding	Clinical interpretation	Citation or search record	Evidence level
		pooled evidence	BE READY, BE SURE, and BE BRIGHT pooled 3-year safety	3,876.4 patient-years		was 0.2/100 patient-years and did not increase with longer exposure.	was low, but active symptomatic IBD was excluded from pivotal trials; trial results therefore do not establish safety in active IBD.	psoriasis: pooled data through 3 years. Br J Dermatol. 2024. doi:10.1093/bjd/ljad429; PMID:37950894.	III and extension
		Case-based literature	Targeted PubMed case-report search	Bimekizumab-treated psoriasis patients	Search through 2026-06-18	No eligible single-patient report of bimekizumab-associated new-onset or exacerbated IBD in psoriasis was identified.	The absence of published cases may reflect recency of exposure and reporting; it should not override the regulatory warning or adjudicated trial events.	PubMed queries: bimekizumab AND inflammatory bowel disease/Crohn/ulcerative colitis AND case report. Search date 2026-06-18.	Negative literature search
	eczema-like / dermatitis-like inflammatory skin events	FDA prescribing information	BIMZELX USPI, Section 6.1	Adult plaque psoriasis, Trials Ps-1 and Ps-2	Placebo-controlled through week 16	Eczema occurred in less than 1% but more than 0.1% of bimekizumab-treated subjects and more frequently than placebo; no dedicated eczematous-eruption warning is present.	The label captures eczema as an uncommon adverse reaction but does not characterize individual-patient inflammatory skin transition.	BIMZELX (bimekizumab-bkzx) US Prescribing Information. UCB; revised 06/2025; FDA SPL set ID 26b88358-871f-4c80-9d80-b2fb16477f81, version 14.	Regulatory label
		Clinical / pooled evidence	Integrated phase II/III psoriasis safety analysis	1,789 patients; 3,109.7 patient-years	Up to 2 years	The pooled publication did not report eczema-like / dermatitis-like inflammatory skin events as a dedicated adverse-event category; eczema was uncommon in the initial label-controlled period.	This domain is better informed by postmarketing reports and event-specific case literature than by aggregate trial coding.	Bimekizumab safety in moderate-to-severe plaque psoriasis. JAMA Dermatol. 2022. doi:10.1001/jamadermatol.2022.1185; PMID:35544084.	Pooled phase II/III; endpoint not dedicated
		Case-based literature	Paradoxical dyshidrosis after bimekizumab	64-year-old woman with recalcitrant psoriasis and	New pruritic vesicular hand/foot dermatitis after	Dyshidrotic eczema developed after near-complete psoriasis clearance. Bimekizumab	This single case supports biological plausibility for a	Paradoxical dyshidrosis following bimekizumab treatment. JAAD Case Rep. 2026. doi:10.1016/j.jdcr.2025.10.062;	Case report

Drug	Safety domain	Evidence stream	Source or study	Population or case	Exposure or onset	Key finding	Clinical interpretation	Citation or search record	Evidence level
				psoriatic arthritis	8 weeks (2 doses)	was continued because of marked benefit; intermittent self-resolving blisters persisted despite topical/systemic attempts.	biologically plausible inflammatory skin transition but cannot estimate frequency or causality.	PMID:41446699; PMCID:PMC12723297.	
		FDA prescribing information	COSENTYX USPI, Sections 5.1 and 6.1	Adult plaque psoriasis placebo-controlled trials	Through week 12	Mucocutaneous Candida infection occurred in 1.2% with secukinumab vs 0.3% with placebo; oral candidiasis occurred in less than 1%. Esophageal and tracheobronchial candidiasis are listed postmarketing.	Candida events are a mechanism-consistent class effect and were less frequent than with dual IL-17A/F blockade in bimekizumab trials.	COSENTYX (secukinumab) US Prescribing Information. Novartis; revised 04/2026; FDA SPL set ID 77c4b13e-7df3-42d4-81db-3d0cddb7f67a, version 29.	Regulatory label
Secukinumab	Candida-related events	Clinical / pooled evidence	Integrated long-term safety across 21 trials	5,181 psoriasis patients; 10,416.9 patient-years	Up to 5 years	Candida infection EAIR in psoriasis was 2.2/100 patient-years; serious infection EAIR was 1.4/100 patient-years.	Long-term trial data support a persistent but relatively low mucocutaneous Candida burden.	Long-term safety of secukinumab: integrated trials and postmarketing surveillance. Arthritis Res Ther. 2019. doi:10.1186/s13075-019-1882-2; PMID:31046809.	Integrated phase II/III and postmarketing
		Case-based literature	Atypical oral candidiasis during secukinumab	50-year-old woman with psoriasis/psoriatic arthritis	Oral lesions reported after approximately 60 days of 300 mg monthly therapy	Cytology and histology confirmed oral candidiasis. Topical miconazole and a 30-day interruption produced resolution; secukinumab was restarted at 150 mg monthly without recurrence during 7 months.	The case illustrates diagnostic difficulty, successful antifungal treatment, and the possibility of dose-adjusted continuation after resolution.	Atypical oral candidiasis during secukinumab. BMC Oral Health. 2021. doi:10.1186/s12903-021-01653-6; PMID:34103043; PMCID:PMC8186152.	Case report
	Inflammatory bowel disease	FDA prescribing information	COSENTYX USPI, Sections 5.4 and 6.1	Plaque psoriasis program: 3,430 exposed patients; 2,725 subject-years	Up to 52 weeks; placebo control through week 12	Three Crohn disease exacerbations, two ulcerative colitis exacerbations, and two new-onset ulcerative colitis cases occurred; no IBD cases occurred among 793 placebo-treated subjects during the	The label advises caution in patients with IBD and monitoring for new or worsening symptoms.	COSENTYX (secukinumab) US Prescribing Information. Novartis; revised 04/2026; FDA SPL set ID 77c4b13e-7df3-42d4-81db-3d0cddb7f67a, version 29.	Regulatory label

Drug	Safety domain	Evidence stream	Source or study	Population or case	Exposure or onset	Key finding	Clinical interpretation	Citation or search record	Evidence level
						12-week controlled period.			
		Clinical / pooled evidence	Retrospective pooled analysis of 21 secukinumab trials	5,181 psoriasis patients within 7,355 patients across indications	16,226.9 patient-years overall	In psoriasis, 14 ulcerative colitis, 5 Crohn disease, and 1 unclassified IBD events were reported (EAIRs 0.13, 0.05, and 0.01/100 patient-years); 14/20 were new onset and annual rates did not increase.	IBD events were uncommon but clinically credible; indication-specific background risk and exclusion criteria complicate cross-drug comparisons.	Incidence rates of IBD with secukinumab across 21 trials. Ann Rheum Dis. 2019. doi:10.1136/annrheumdis-2018-214273; PMID:30674475.	Pooled clinical trials
		Case-based literature	Secukinumab-associated Crohn disease	54-year-old woman with plaque psoriasis and psoriatic arthritis; family history of ulcerative colitis	Symptoms began after 14 months	Ileal Crohn disease was diagnosed. Secukinumab withdrawal improved bowel symptoms; subsequent tildrakizumab controlled skin, joint, and bowel manifestations.	The case highlights potential susceptibility factors and a dechallenge response, while psoriasis itself confers background IBD risk.	Secukinumab-induced inflammatory bowel disease. Case Rep Med. 2020. doi:10.1155/2020/9404505; PMID:32774388; PMCID:PMC7395993.	Case report
	eczema-like / dermatitis-like inflammatory skin events	FDA prescribing information	COSENTYX USPI, Section 5.5	Postmarketing secukinumab exposure	Onset from days to months	Severe atopic dermatitis-like eruptions, dyshidrotic eczema, and erythroderma have been reported; some required hospitalization. Discontinuation may be needed, although some patients continued therapy with eczema treatment.	eczema-like / dermatitis-like inflammatory skin events is an explicit postmarketing warning and should be considered separately from broad dermatitis PTs.	COSENTYX (secukinumab) US Prescribing Information. Novartis; revised 04/2026; FDA SPL set ID 77c4b13e-7df3-42d4-81db-3d0cddb7f67a, version 29.	Regulatory label
		Clinical / pooled evidence	Anti-IL-17A eczematous-eruption cohort	468 psoriasis patients starting secukinumab or ixekizumab; 27 eruptions	Mean onset 16.9 +/- 17.0 weeks	Eruptions occurred in 5.8%: acute eczema 40.7%, atopic dermatitis-like rash 40.7%, and psoriasiform eruption 18.5%; all 12 biopsied cases showed spongiosis.	This real-world case series provides event-specific evidence that routine trial AE coding may under-recognize paradoxical	Clinical and histopathological characterization of anti-IL-17 eruptions. Expert Opin Biol Ther. 2020. doi:10.1080/14712598.2020.1727439; PMID:32045273.	Retrospective case series

Drug	Safety domain	Evidence stream	Source or study	Population or case	Exposure or onset	Key finding	Clinical interpretation	Citation or search record	Evidence level
							eczema.		
		Case-based literature	Generalized eczematous eruption after secukinumab	20-year-old man with severe psoriasis	New lesions 2 months after initiation	Biopsy showed spongiotic dermatitis. Lesions progressed despite topical/systemic corticosteroids while secukinumab continued; withdrawal plus cyclosporine/prednisone produced marked improvement within 2 weeks.	The dechallenge response and histology support a eczema-like / dermatitis-like presentation, but evidence remains single-patient.	Treatment of paradoxical eczematous eruption with secukinumab. Medicine. 2023. doi:10.1097/MD.0000000000032844; PMID:36820537; PMCID:PMC9907985.	Case report
Ixekizumab	Candida-related events	FDA prescribing information	TALTZ USPI, Sections 5.1, 6.1, and 6.3	Adult plaque psoriasis trials and postmarketing exposure	Controlled induction through week 12	Oral candidiasis occurred more often than placebo but at less than 1%; postmarketing reports include esophageal and disseminated Candida-related events.	The label supports Candida surveillance, including dysphagia as a clue to esophageal disease.	TALTZ (ixekizumab) US Prescribing Information. Eli Lilly; revised 11/2024; FDA SPL set ID ac96658a-d7dc-4c7c-8928-2adcdf4318b2, version 39.	Regulatory label
		Clinical / pooled evidence	Integrated safety from 13 psoriasis studies	5,898 patients; 17,003.4 patient-years	Up to 5 years	Candidiasis occurred in 327 patients (IR 1.9/100 patient-years), predominantly mucocutaneous; oral candidiasis occurred in 140 (IR 0.8). One esophageal event led to discontinuation; no deep-organ or bloodstream Candida infection was reported.	Long-term rates were stable or declined after year 1 and provide a quantitative benchmark for FAERS findings.	Safety of ixekizumab through 5 years. Dermatol Ther (Heidelb). 2020. doi:10.1007/s13555-019-00340-3; PMID:31749092; PMCID:PMC6994584.	Integrated clinical trials
		Case-based literature	Esophageal candidiasis during ixekizumab	55-year-old man with plaque psoriasis	Dysphagia after 18 weeks	Endoscopy confirmed esophageal candidiasis. Ixekizumab was suspended and itraconazole cleared infection; retreatment restored PASI100 without recurrence through week 40.	The case supports temporary interruption and successful rechallenge after documented resolution in a selected patient.	Esophageal candidiasis in an ixekizumab-treated psoriasis patient. Dermatol Ther. 2022. doi:10.1111/dth.15361; PMID:35137503; PMCID:PMC9286677.	Case report
	Inflammatory bowel disease	FDA prescribing information	TALTZ USPI, Section 5.5 and plaque psoriasis	Adult plaque psoriasis trials	12-week placebo-controlled period	Crohn disease (0.1%) and ulcerative colitis (0.2%), including exacerbations, occurred more often with	Even low absolute trial frequency warrants	TALTZ (ixekizumab) US Prescribing Information. Eli Lilly; revised 11/2024; FDA SPL set ID ac96658a-d7dc-4c7c-8928-2adcdf4318b2, version 39.	Regulatory label

Drug	Safety domain	Evidence stream	Source or study	Population or case	Exposure or onset	Key finding	Clinical interpretation	Citation or search record	Evidence level
			safety			ixekizumab than placebo (0%). The label directs discontinuation and medical management if IBD develops.	symptom screening because severe new-onset disease is represented in case literature.		
		Clinical / pooled evidence	Integrated safety from 13 psoriasis studies	5,898 patients; 17,003.4 patient-years	Up to 5 years	Narrow IBD events occurred in 26 patients (0.4%; IR 0.2/100 patient-years): 18 ulcerative colitis and 7 Crohn disease; adjudicated annual rates did not increase over time.	The low stable EAIR contextualizes spontaneous reports but does not exclude severe susceptible-patient events.	Safety of ixekizumab through 5 years. Dermatol Ther (Heidelb). 2020. doi:10.1007/s13555-019-00340-3; PMID:31749092; PMCID:PMC6994584.	Integrated clinical trials
		Case-based literature	New-onset Crohn disease after ixekizumab	48-year-old woman with chronic plaque psoriasis	Approximately 12 weeks after initiation	Ileal Crohn disease developed; ixekizumab was stopped, budesonide used as a bridge, and adalimumab subsequently controlled Crohn disease, psoriasis, and psoriatic arthritis.	Temporal association and improvement after withdrawal support vigilance, but baseline psoriasis-associated IBD risk remains a confounder.	Ixekizumab-associated new-onset IBD. ACG Case Rep J. 2020. doi:10.14309/crj.0000000000000316; PMID:32440523; PMCID:PMC7209798.	Case report
	eczema-like / dermatitis-like inflammatory skin events	FDA prescribing information	TALTZ USPI, Section 5.4	Postmarketing ixekizumab exposure	Onset from days to months	Severe atopic dermatitis-like eruptions, dyshidrotic eczema, and erythroderma were reported; some required hospitalization and discontinuation, although selected patients continued with eczema treatment.	This is an explicit postmarketing warning and directly supports a narrow narrow eczema-like / dermatitis-like endpoint.	TALTZ (ixekizumab) US Prescribing Information. Eli Lilly; revised 11/2024; FDA SPL set ID ac96658a-d7dc-4c7c-8928-2adcdf4318b2, version 39.	Regulatory label
		Clinical / pooled evidence	Integrated safety from 13 psoriasis studies	5,898 patients; 17,003.4 patient-years	Up to 5 years	Eczema was reported at 1.0/100 patient-years; one worsening eczematous reaction was considered a possible paradoxical event, resolved, and treatment continued.	Aggregate eczema coding includes heterogeneous conditions; event-specific postmarketing adjudication	Safety of ixekizumab through 5 years. Dermatol Ther (Heidelb). 2020. doi:10.1007/s13555-019-00340-3; PMID:31749092; PMCID:PMC6994584.	Integrated clinical trials

Drug	Safety domain	Evidence stream	Source or study	Population or case	Exposure or onset	Key finding	Clinical interpretation	Citation or search record	Evidence level
							remains necessary.		
		Case-based literature	Atopic dermatitis-like phenotypic shift during ixekizumab	Patient with longstanding plaque psoriasis after prolonged secukinumab and ixekizumab control	Long-term IL-17A inhibitor exposure	A de novo eczematous eruption developed; biopsy showed spongiotic dermatitis. Upadacitinib subsequently controlled both psoriasis and atopic dermatitis-like disease.	The case illustrates immune-axis redistribution and the need to distinguish true eczema from psoriasis flare or nonspecific dermatitis.	Phenotypic Shift during Treatment of Plaque Psoriasis with Ixekizumab. J Clin Aesthet Dermatol. 2024; PMID:39758219; PMCID:PMC11694639.	Case report

Evidence streams were used for clinical contextualization and should not be interpreted as incidence estimates or causal validation of spontaneous-report signals. Skin-event terminology was kept descriptive and aligned with the prespecified eczema-like / dermatitis-like endpoint definitions.

Supplementary Table S8. Label-anchored frequency context for mapped study endpoints.

Drug	Target selectivity	Study safety domain	FDA label source	Labelled adverse event or group	Author-mapped label frequency category	Author-mapped frequency range	Mapped study endpoint	Interpretation for current analysis
Bimekizumab	Dual IL-17A/F inhibition	Candida-related events	BIMZELX US FDA prescribing information; accessed 2026-06-26	Oral/oropharyngeal candidiasis	Common	1% to <10%	Oral candidiasis; Any Candida-related event	Higher labelled frequency category than selective IL-17A comparators, supporting label-concordant interpretation of the bimekizumab Candida-related signal.
				Vulvovaginal candidiasis or vulvovaginal mycotic infection	Common	1% to <10%	Any Candida-related event; site-specific Candida-related PTs	Supports a broader Candida-related benchmark for bimekizumab beyond oral events.
				Mucosal and skin candidiasis, including oesophageal candidiasis	Uncommon	0.1% to <1%	Any Candida-related event; oesophageal candidiasis	Supports interpretation of oesophageal candidiasis as a lower-frequency but label-recognized manifestation.
		Inflammatory bowel disease		Inflammatory bowel disease	Uncommon	0.1% to <1%	IBD composite; ulcerative colitis; Crohn's disease where applicable	Places IBD in the shared IL-17-inhibitor warning context; the study tests whether reporting is additionally enriched for bimekizumab versus selective IL-17A comparators.
		Eczema-like / dermatitis-like events		Rash, dermatitis and eczema	Common	1% to <10%	Eczema-like / dermatitis-like endpoint where PTs map to the prespecified dictionary; nonspecific rash terms are contextual	Shows that labelled cutaneous events are common for bimekizumab, but these terms are broader than the prespecified eczema-like / dermatitis-like endpoint.
				Acne	Common	1% to <10%	Contextual cutaneous label term; not part of the prespecified eczema-like / dermatitis-like endpoint	Provides cutaneous label context but should not be interpreted as evidence for a stable eczema-like / dermatitis-like inflammatory skin signal.
Secukinumab	Selective IL-17A inhibition	Candida-related events	COSENTYX US FDA prescribing information; accessed 2026-06-26	Oral candidiasis	Uncommon	0.1% to <1%	Oral candidiasis; Any Candida-related event	Provides a lower-frequency comparator benchmark for oral candidiasis.
				Mucosal and skin candidiasis, including oesophageal candidiasis	Frequency not known	Not estimable from label	Any Candida-related event; oesophageal candidiasis	Listed in the regulatory safety context without an estimable frequency; should not be interpreted as absence of risk.
		Inflammatory bowel disease		Inflammatory bowel disease	Uncommon	0.1% to <1%	IBD composite; ulcerative colitis; Crohn's disease where applicable	Places IBD in the shared IL-17-inhibitor warning context for comparator interpretation.
		Eczema-like / dermatitis-like events		Dermatitis, including eczema	Common	1% to <10%	Eczema-like / dermatitis-like endpoint where PTs map to the prespecified dictionary	Shows that eczema/dermatitis is also labelled for a selective IL-17A comparator, supporting cautious interpretation of eczema-like / dermatitis-like inflammatory skin signals.
				Urticaria	Uncommon	0.1% to <1%	Contextual cutaneous/hypersensitivity label term; not specific to the eczema-like / dermatitis-like endpoint	Provides cutaneous label context but is not specific to the prespecified eczema-like / dermatitis-like endpoint.

Drug	Target selectivity	Study safety domain	FDA label source	Labelled adverse event or group	Author-mapped label frequency category	Author-mapped frequency range	Mapped study endpoint	Interpretation for current analysis
Ixekizumab	Selective IL-17A inhibition		TALTZ US FDA prescribing information; accessed 2026-06-26	Dyshidrotic eczema	Uncommon	0.1% to <1%	Eczema-like / dermatitis-like endpoint where PTs map to the prespecified dictionary	Provides a comparator label benchmark for an eczematous term overlapping the study domain.
				Exfoliative dermatitis	Rare	0.01% to <0.1%	Contextual severe cutaneous label term; not specific to the eczema-like / dermatitis-like endpoint	Provides rare severe cutaneous label context without implying equivalence to the prespecified eczema-like / dermatitis-like endpoint.
				Pyoderma gangrenosum, generalized exfoliative dermatitis, angioedema	Frequency not known	Not estimable from label	Contextual postmarketing cutaneous/hypersensitivity label terms; not directly equivalent to the prespecified endpoint	Postmarketing terms with unestimable frequency provide qualitative safety context only.
		Candida-related events		Oral candidiasis	Uncommon	0.1% to <1%	Oral candidiasis; Any Candida-related event	Provides a lower-frequency comparator benchmark for oral candidiasis.
				Oesophageal candidiasis	Rare	0.01% to <0.1%	Oesophageal candidiasis; Any Candida-related event	Supports interpretation of oesophageal candidiasis as rare in the selective IL-17A comparator label context.
		Inflammatory bowel disease		Inflammatory bowel disease	Uncommon	0.1% to <1%	IBD composite; ulcerative colitis; Crohn's disease where applicable	Places IBD in the shared IL-17-inhibitor warning context for comparator interpretation.
		Eczema-like / dermatitis-like events		Urticaria	Uncommon	0.1% to <1%	Contextual cutaneous/hypersensitivity label term; not specific to the eczema-like / dermatitis-like endpoint	Provides cutaneous label context but is not specific to the prespecified eczema-like / dermatitis-like endpoint.
				Rash and eczema	Uncommon	0.1% to <1%	Eczema-like / dermatitis-like endpoint where PTs map to the prespecified dictionary; nonspecific rash terms are contextual	Provides comparator label context for rash/eczema while preserving the distinction between nonspecific rash and the prespecified eczema-like / dermatitis-like endpoint.

Frequency categories were author-mapped from US prescribing information for contextual interpretation. Label frequency categories are not directly comparable with FAERS/JADER reporting odds ratios.

Supplementary Table S9. Final analysis-set characteristics by drug and database.

Section	Characteristic	Category	FAERS				JADER			
			Bimekizumab	Secukinumab	Ixekizumab	Pooled comparator	Bimekizumab	Secukinumab	Ixekizumab	Pooled comparator
Analysis set	Final psoriasis-associated reports/cases	All	7,850 (100.0%)	6,623 (100.0%)	4,251 (100.0%)	10,971 (100.0%)	104 (100.0%)	707 (100.0%)	175 (100.0%)	890 (100.0%)
Age	Age, years	Median (IQR)	52.0 (41.0-61.0)	55.0 (43.0-65.0)	54.0 (40.0-63.0)	54.0 (41.0-64.0)	-	-	-	-
	Age category	<20 years	24 (0.3%)	116 (1.8%)	183 (4.3%)	299 (2.7%)	0 (0.0%)	11 (1.6%)	0 (0.0%)	11 (1.2%)
		20-39 years	1,053 (13.4%)	642 (9.7%)	685 (16.1%)	1,332 (12.1%)	12 (11.5%)	49 (6.9%)	16 (9.1%)	65 (7.3%)
		40-59 years	2,389 (30.4%)	1,519 (22.9%)	1,401 (33.0%)	2,957 (27.0%)	29 (27.9%)	186 (26.3%)	65 (37.1%)	256 (28.8%)
		60-79 years	1,372 (17.5%)	1,413 (21.3%)	1,126 (26.5%)	2,559 (23.3%)	43 (41.3%)	257 (36.4%)	61 (34.9%)	318 (35.7%)
		>=80 years	76 (1.0%)	130 (2.0%)	81 (1.9%)	214 (2.0%)	9 (8.7%)	51 (7.2%)	17 (9.7%)	69 (7.8%)
		Unknown/missing	2,936 (37.4%)	2,803 (42.3%)	775 (18.2%)	3,610 (32.9%)	11 (10.6%)	153 (21.6%)	16 (9.1%)	171 (19.2%)
Sex	Sex	Female	1,411 (18.0%)	3,682 (55.6%)	2,470 (58.1%)	6,193 (56.4%)	33 (31.7%)	253 (35.8%)	69 (39.4%)	325 (36.5%)
		Male	1,025 (13.1%)	2,610 (39.4%)	1,538 (36.2%)	4,188 (38.2%)	69 (66.3%)	446 (63.1%)	93 (53.1%)	544 (61.1%)
		Other/unknown	0 (0.0%)	1 (0.0%)	1 (0.0%)	2 (0.0%)	0 (0.0%)	1 (0.1%)	0 (0.0%)	1 (0.1%)
		Unknown/missing	5,414 (69.0%)	330 (5.0%)	242 (5.7%)	588 (5.4%)	2 (1.9%)	7 (1.0%)	13 (7.4%)	20 (2.2%)
Reporter	Reporter qualification	Physician	526 (6.7%)	1,595 (24.1%)	178 (4.2%)	1,812 (16.5%)	93 (89.4%)	644 (91.1%)	138 (78.9%)	790 (88.8%)
		Pharmacist	74 (0.9%)	92 (1.4%)	66 (1.6%)	158 (1.4%)	8 (7.7%)	16 (2.3%)	8 (4.6%)	24 (2.7%)
		Other health professional	913 (11.6%)	1,264 (19.1%)	423 (10.0%)	1,722 (15.7%)	2 (1.9%)	39 (5.5%)	1 (0.6%)	40 (4.5%)
		Consumer	1,203 (15.3%)	2,722 (41.1%)	2,481 (58.4%)	5,222 (47.6%)	1 (1.0%)	8 (1.1%)	28 (16.0%)	36 (4.0%)
		Lawyer	0 (0.0%)	0 (0.0%)	1 (0.0%)	1 (0.0%)	0 (0.0%)	0 (0.0%)	0 (0.0%)	0 (0.0%)
		Other/unknown	0 (0.0%)	0 (0.0%)	0 (0.0%)	0 (0.0%)	0 (0.0%)	0 (0.0%)	0 (0.0%)	0 (0.0%)
		Unknown/missing	5,134 (65.4%)	950 (14.3%)	1,102 (25.9%)	2,056 (18.7%)	0 (0.0%)	0 (0.0%)	0 (0.0%)	0 (0.0%)
Geography	Reporter country/geography	United States	6,690 (85.2%)	3,777 (57.0%)	3,704 (87.1%)	7,530 (68.6%)	0 (0.0%)	0 (0.0%)	0 (0.0%)	0 (0.0%)
		Japan	80 (1.0%)	72 (1.1%)	38 (0.9%)	116 (1.1%)	0 (0.0%)	0 (0.0%)	0 (0.0%)	0 (0.0%)
		Canada	536 (6.8%)	966 (14.6%)	36 (0.8%)	1,013 (9.2%)	0 (0.0%)	0 (0.0%)	0 (0.0%)	0 (0.0%)
		United Kingdom	172 (2.2%)	361 (5.5%)	25 (0.6%)	386 (3.5%)	0 (0.0%)	0 (0.0%)	0 (0.0%)	0 (0.0%)
		France	31 (0.4%)	65 (1.0%)	57 (1.3%)	130 (1.2%)	0 (0.0%)	0 (0.0%)	0 (0.0%)	0 (0.0%)
		Germany	64 (0.8%)	113 (1.7%)	49 (1.2%)	165 (1.5%)	0 (0.0%)	0 (0.0%)	0 (0.0%)	0 (0.0%)
		Other countries	201 (2.6%)	1,181 (17.8%)	308 (7.2%)	1,508 (13.7%)	0 (0.0%)	0 (0.0%)	0 (0.0%)	0 (0.0%)
		Japan (database scope)	0 (0.0%)	0 (0.0%)	0 (0.0%)	0 (0.0%)	104 (100.0%)	707 (100.0%)	175 (100.0%)	890 (100.0%)

Section	Characteristic	Category	FAERS				JADER			
			**Bimekizumab**	**Secukinumab**	**Ixekizumab**	**Pooled comparator**	**Bimekizumab**	**Secukinumab**	**Ixekizumab**	**Pooled comparator**
Reporting period	Report year	<2023	0 (0.0%)	0 (0.0%)	0 (0.0%)	0 (0.0%)	10 (9.6%)	608 (86.0%)	120 (68.6%)	733 (82.4%)
		2023	52 (0.7%)	1,640 (24.8%)	580 (13.6%)	2,230 (20.3%)	22 (21.2%)	55 (7.8%)	26 (14.9%)	81 (9.1%)
		2024	1,271 (16.2%)	2,901 (43.8%)	2,223 (52.3%)	5,172 (47.1%)	38 (36.5%)	27 (3.8%)	17 (9.7%)	45 (5.1%)
		2025	5,285 (67.3%)	1,705 (25.7%)	1,324 (31.1%)	3,065 (27.9%)	34 (32.7%)	17 (2.4%)	12 (6.9%)	31 (3.5%)
		2026	1,242 (15.8%)	377 (5.7%)	124 (2.9%)	504 (4.6%)	0 (0.0%)	0 (0.0%)	0 (0.0%)	0 (0.0%)
		Unknown/missing	0 (0.0%)	0 (0.0%)	0 (0.0%)	0 (0.0%)	0 (0.0%)	0 (0.0%)	0 (0.0%)	0 (0.0%)
Outcome coding	Any serious-outcome coding	Yes	2,916 (37.1%)	3,702 (55.9%)	940 (22.1%)	4,714 (43.0%)	0 (0.0%)	0 (0.0%)	0 (0.0%)	0 (0.0%)
		No	4,934 (62.9%)	2,921 (44.1%)	3,311 (77.9%)	6,257 (57.0%)	0 (0.0%)	0 (0.0%)	0 (0.0%)	0 (0.0%)
		Not harmonised	0 (0.0%)	0 (0.0%)	0 (0.0%)	0 (0.0%)	104 (100.0%)	707 (100.0%)	175 (100.0%)	890 (100.0%)
	Hospitalisation coding	Yes	825 (10.5%)	1,302 (19.7%)	355 (8.4%)	1,682 (15.3%)	0 (0.0%)	0 (0.0%)	0 (0.0%)	0 (0.0%)
		No	7,025 (89.5%)	5,321 (80.3%)	3,896 (91.6%)	9,289 (84.7%)	0 (0.0%)	0 (0.0%)	0 (0.0%)	0 (0.0%)
		Not harmonised	0 (0.0%)	0 (0.0%)	0 (0.0%)	0 (0.0%)	104 (100.0%)	707 (100.0%)	175 (100.0%)	890 (100.0%)
	Death coding	Yes	72 (0.9%)	203 (3.1%)	70 (1.6%)	280 (2.6%)	0 (0.0%)	0 (0.0%)	0 (0.0%)	0 (0.0%)
		No	7,778 (99.1%)	6,420 (96.9%)	4,181 (98.4%)	10,691 (97.4%)	0 (0.0%)	0 (0.0%)	0 (0.0%)	0 (0.0%)
		Not harmonised	0 (0.0%)	0 (0.0%)	0 (0.0%)	0 (0.0%)	104 (100.0%)	707 (100.0%)	175 (100.0%)	890 (100.0%)

Note: Values are n (%) unless otherwise indicated. Pooled comparator columns include secukinumab, ixekizumab, and rare reports/cases containing both; these mixed records were not assigned to either single-drug column. Exact age was available in FAERS, whereas JADER age was recorded in decade bands. JADER cases before 2023 were grouped as <2023; the FAERS analysis window began in 2023Q4. JADER outcome fields were not harmonised with FAERS OUTC fields.

Supplementary Table S9b. Age-band distribution in FAERS and JADER by study group.

Panel A. FAERS

Age band	Bimekizumab, n (%)	Selective IL-17A comparator, n (%)
<20 years	24 (0.3%)	299 (2.7%)
20-39 years	1,053 (13.4%)	1,332 (12.1%)
40-59 years	2,389 (30.4%)	2,957 (27.0%)
60-79 years	1,372 (17.5%)	2,559 (23.3%)
>=80 years	76 (1.0%)	214 (2.0%)
Unknown/missing	2,936 (37.4%)	3,610 (32.9%)

Panel B. JADER

Age band	Bimekizumab, n (%)	Selective IL-17A comparator, n (%)
<20 years	0 (0.0%)	11 (1.2%)
20-39 years	12 (11.5%)	65 (7.3%)
40-59 years	29 (27.9%)	256 (28.8%)
60-79 years	43 (41.3%)	318 (35.7%)
>=80 years	9 (8.7%)	69 (7.8%)
Unknown/missing	11 (10.6%)	171 (19.2%)

Note: Percentages are calculated within each database–drug stratum using the full analytic denominator; unknown/missing age is retained as a separate category.

Supplementary Table S9c. Complete-case Candida-related reporting contrasts stratified by sex and age.

Database	Stratification	Stratum	Bimekizumab events/total	Comparator events/total	ROR (95% CI)
FAERS	Sex	Female	138/1,411	89/6,193	7.43 (5.66–9.77)
		Male	70/1,025	39/4,188	7.80 (5.24–11.61)
JADER	Sex	Female	5/33	12/325	4.66 (1.53–14.17)
		Male	13/69	12/544	10.29 (4.48–23.64)
FAERS	Age	<65 years	234/4,113	69/5,582	4.82 (3.67–6.32)
		>=65 years	58/801	22/1,779	6.23 (3.79–10.26)

Note: RORs are complete-case, exploratory relative reporting contrasts and are unadjusted. Estimates were reported only when both event-positive cells contained at least five reports/cases. Comparator denotes pooled selective IL-17A inhibitors.

Supplementary Table S10. Ten most frequently reported Preferred Terms by drug and database.

Panel A. FAERS

Drug	Rank	Preferred Term	Reports/cases with PT, n/N (%)
Bimekizumab	1	Psoriasis	1,791/7,850 (22.8%)
	2	Product dose omission issue	1,408/7,850 (17.9%)
	3	Drug ineffective	686/7,850 (8.7%)
	4	Off label use	546/7,850 (7.0%)
	5	Psoriatic arthropathy	497/7,850 (6.3%)
	6	Inappropriate schedule of product administration	486/7,850 (6.2%)
	7	Product availability issue	475/7,850 (6.1%)
	8	Therapy interrupted	461/7,850 (5.9%)
	9	Injection site pain	458/7,850 (5.8%)
	10	Incorrect dose administered	320/7,850 (4.1%)
Secukinumab	1	Psoriasis	1,969/6,623 (29.7%)
	2	Drug ineffective	1,130/6,623 (17.1%)
	3	Pruritus	795/6,623 (12.0%)
	4	Pain	699/6,623 (10.6%)
	5	Arthralgia	697/6,623 (10.5%)
	6	Therapeutic product effect incomplete	575/6,623 (8.7%)
	7	Skin exfoliation	545/6,623 (8.2%)
	8	Psoriatic arthropathy	484/6,623 (7.3%)
	9	Malaise	445/6,623 (6.7%)
	10	Fatigue	433/6,623 (6.5%)
Ixekizumab	1	Psoriasis	554/4,251 (13.0%)
	2	Drug ineffective	246/4,251 (5.8%)
	3	Incorrect dose administered	217/4,251 (5.1%)
	4	Injection site reaction	208/4,251 (4.9%)
	5	Therapy interrupted	198/4,251 (4.7%)
	6	Injection site pain	179/4,251 (4.2%)
	7	Injection site erythema	178/4,251 (4.2%)
	8	COVID-19	129/4,251 (3.0%)
	9	Inappropriate schedule of product administration	127/4,251 (3.0%)
	10	Injection site swelling	126/4,251 (3.0%)
Pooled selective IL-17A comparator	1	Psoriasis	2,550/10,971 (23.2%)
	2	Drug ineffective	1,403/10,971 (12.8%)
	3	Pruritus	852/10,971 (7.8%)
	4	Arthralgia	747/10,971 (6.8%)
	5	Pain	741/10,971 (6.8%)
	6	Therapeutic product effect incomplete	597/10,971 (5.4%)
	7	Psoriatic arthropathy	570/10,971 (5.2%)
	8	Skin exfoliation	555/10,971 (5.1%)
	9	Nasopharyngitis	527/10,971 (4.8%)
	10	Inappropriate schedule of product administration	519/10,971 (4.7%)

Panel B. JADER

Drug	Rank	Preferred Term	Reports/cases with PT, n/N (%)
Bimekizumab	1	食道カンジダ症	17/104 (16.3%)
	2	乾癬性関節症	4/104 (3.8%)
	3	間質性肺疾患	4/104 (3.8%)
	4	クローン病	3/104 (2.9%)
	5	汎血球減少症	3/104 (2.9%)
	6	下痢	2/104 (1.9%)
	7	入院	2/104 (1.9%)
	8	口腔カンジダ症	2/104 (1.9%)
	9	帯状疱疹	2/104 (1.9%)
	10	心不全	2/104 (1.9%)

Drug	Rank	Preferred Term	Reports/cases with PT, n/N (%)
Secukinumab	1	間質性肺疾患	45/707 (6.4%)
	2	蜂巣炎	39/707 (5.5%)
	3	乾癬性関節症	23/707 (3.3%)
	4	潰瘍性大腸炎	23/707 (3.3%)
	5	食道カンジダ症	19/707 (2.7%)
	6	肺炎	18/707 (2.5%)
	7	丹毒	14/707 (2.0%)
	8	炎症性腸疾患	11/707 (1.6%)
	9	腎機能障害	11/707 (1.6%)
	10	リンパ節転移	10/707 (1.4%)
Ixekizumab	1	蜂巣炎	13/175 (7.4%)
	2	間質性肺疾患	12/175 (6.9%)
	3	炎症性腸疾患	6/175 (3.4%)
	4	潰瘍性大腸炎	4/175 (2.3%)
	5	細菌性関節炎	4/175 (2.3%)
	6	肺炎	4/175 (2.3%)
	7	アナフィラキシーショック	3/175 (1.7%)
	8	アナフィラキシー反応	3/175 (1.7%)
	9	咽頭炎	3/175 (1.7%)
	10	直腸癌	3/175 (1.7%)
Pooled selective IL-17A comparator	1	間質性肺疾患	57/890 (6.4%)
	2	蜂巣炎	52/890 (5.8%)
	3	潰瘍性大腸炎	27/890 (3.0%)
	4	乾癬性関節症	24/890 (2.7%)
	5	肺炎	22/890 (2.5%)
	6	食道カンジダ症	20/890 (2.2%)
	7	炎症性腸疾患	19/890 (2.1%)
	8	丹毒	15/890 (1.7%)
	9	細菌性関節炎	13/890 (1.5%)
	10	細菌性肺炎	12/890 (1.3%)

Note: The top 10 Preferred Terms are presented as descriptive reporting context and were not used for comprehensive signal ranking. Reporting proportion is the number of reports/cases containing the PT divided by the total reports/cases in that database–drug stratum. JADER PT labels are retained as provided in the source output.

Supplementary Table S11. Candida-related PT-level threshold summary in FAERS and JADER.

Panel A. FAERS

Preferred Term	Bimekizumab positive, n11	Comparator positive	ROR (95% CI)	n11 ≥ 5	n11 ≥ 10	Three-metric signal
Candida infection	173	36	6.84 (4.77–9.81)	Yes	Yes	Yes
Oropharyngeal candidiasis	4	6	0.93 (0.26–3.30)	No	No	No
Vulvovaginal candidiasis	9	15	0.84 (0.37–1.92)	Yes	No	No
Oral candidiasis	292	66	6.38 (4.88–8.35)	Yes	Yes	Yes
Oesophageal candidiasis	69	13	7.47 (4.13–13.53)	Yes	Yes	Yes
Gastrointestinal candidiasis	3	3	1.40 (0.28–6.93)	No	No	No
Genital candidiasis	4	4	1.40 (0.35–5.59)	No	No	No
Nasal candidiasis	1	0	4.19 (0.17–102.95)	No	No	No
Skin candida	13	2	9.10 (2.05–40.33)	Yes	Yes	No
Mucocutaneous candidiasis	1	1	1.40 (0.09–22.35)	No	No	No
Systemic candida	1	0	4.19 (0.17–102.95)	No	No	No
Candida sepsis	1	1	1.40 (0.09–22.35)	No	No	No
Chronic hyperplastic candidiasis	0	1	0.47 (0.02–11.44)	No	No	No

Panel B. JADER

Preferred Term	Bimekizumab positive, n11	Comparator positive	ROR (95% CI)	n11 ≥ 5	n11 ≥ 10	Three-metric signal
口腔カンジダ症	2	5	3.47 (0.66–18.12)	No	No	No
食道カンジダ症	17	20	8.50 (4.29–16.83)	Yes	Yes	Yes
気管カンジダ症	0	1	2.84 (0.11–70.10)	No	No	No
中咽頭カンジダ症	0	1	2.84 (0.11–70.10)	No	No	No
カンジダ感染	0	2	1.70 (0.08–35.66)	No	No	No

Note: n11 denotes bimekizumab reports/cases containing the PT. The primary PT interpretation threshold was n11 ≥ 5; PTs with n11 < 5 were descriptive only. n11 ≥ 10 was examined as a sensitivity threshold. "Three-metric signal" reflects concordant source-output criteria across ROR, PRR, and IC. Comparator denotes pooled selective IL-17A inhibitors. The complete 4,256-row PT-level threshold audit is provided separately as Supplementary Data 2. JADER PT labels are retained as provided in the source output.

Supplementary Table S12. Evaluability and sensitivity analyses for reported time to onset of Candida-related events in FAERS.

Analysis	Group	Evaluable, n	Missing/unevaluable, n/N (%)	Median TTO, days (IQR)	≤30 days, n (%)	≤90 days, n (%)
Primary strict-date TTO	Bimekizumab	74	440/514 (85.6%)	26.5 (4–68)	38 (51.4%)	62 (83.8%)
	Selective IL-17A comparator	45	94/139 (67.6%)	258 (67–372)	9 (20.0%)	16 (35.6%)
Common calendar window (2023-10-18 to 2026-03-31)	Bimekizumab	55	—	24 (0–64)	30 (54.5%)	48 (87.3%)
	Selective IL-17A comparator	5	—	89 (7–98)	2 (40.0%)	3 (60.0%)

Note: For the primary strict-date TTO analysis, missing/unevaluable values use all Candida-positive FAERS reports as the denominator. For the common-calendar-window sensitivity analysis, only evaluable counts and timing summaries were provided in the source output. Percentages within 30 and 90 days use evaluable reports as the denominator. Comparator denotes pooled selective IL-17A inhibitors. TTO, time to onset; IQR, interquartile range.
